# The global, regional, and national burden of non-rheumatic degenerative mitral valve disease from 1990 to 2021 and forecast for 2050

**DOI:** 10.3389/fcvm.2026.1583290

**Published:** 2026-01-28

**Authors:** Ke Si, Yucheng Hou, Jun Wang, Guijun Huo, Cheng Sun

**Affiliations:** 1 Department of Cardiovascular Surgery, The First Affiliated Hospital of Soochow University, Suzhou, China; 2 Suzhou Medical College, Soochow University, Suzhou, China; 3 Department of Vascular Surgery, The Affiliated Suzhou Hospital of Nanjing Medical University, Suzhou Municipal Hospital, Suzhou, China; 4 Department of Cardiovascular Surgery, National Center for Cardiovascular Diseases and Fuwai Hospital, Chinese Academy of Medical Sciences, Peking Union Medical College, Beijing, China

**Keywords:** disability-adjusted life years, estimated annual percentage changes, global burden disease, sociodemographic index, non-rheumatic degenerative mitral valve disease

## Abstract

**Background:**

Given the changing epidemiological and burden trends of non-rheumatic degenerative mitral valve disease (DMVD), it is crucial to re-examine geographical differences and trends. Here, we describe the current trends of DMVD epidemiology using data from the Global Burden of Diseases (GBD) study from 1990 to 2021, with forecasts extending to 2050.

**Methods:**

Annual case numbers and age-standardized rates (ASR) of incidence, prevalence, deaths, and disability-adjusted life years (DALYs) for DMVD between 1990 and 2021, as well as their estimated annual percentage changes (EAPC), were derived from the 2021 GBD study. Exponential smoothing and autoregressive integrated moving average models are employed for forecasting the future trends of DMVD.

**Results:**

Between 1990 and 2021, the number of DMVD incidence cases increased from 566,261 (95% uncertainty interval [UI]: 523,330–609,607) to 1,162,558 (1,084,358–1,244,874), corresponding to a 105.3% (77.9–137.9) rise. Additionally, DMVD-related death cases rose by 53.8% (21.2–97.7), from 23,954 (21,032–26,296) to 36,844 (31,883–41,572). However, there is a decline in ASR of incidence and death. Stratified analysis revealed that the age-standardized incidence rate (ASIR) in males is nearly twice that of females, while the age-standardized death rate (ASDR) is higher in females. These trends are not expected to improve significantly by 2050. Concurrently, the ASIR peaks in individuals aged 65–69 and 70–74. In 2021, higher socio-demographic index (SDI) countries bore a greater DMVD burden, while lower-SDI countries faced greater cross-country inequalities.

**Conclusions:**

The number of DMVD epidemiological indicators is rising annually. Higher-SDI countries need to adjust healthcare policies to address aging populations and support lower-SDI countries with medical resources and cardiology expertise. This study suggests that age 65 is a meaningful threshold for screening. Additionally, Our findings advocate for stratified public health interventions: incidence-based screening targeting males and mortality-focused treatment intensification for females.

## Introduction

Non-rheumatic degenerative mitral valve disease (DMVD) is characterized by age-related degenerative changes in the mitral valve's connective tissue, manifesting as myxomatous degeneration, leading to regurgitation or prolapse (1). Hemodynamic alterations associated with moderate to severe mitral stenosis or regurgitation are frequently implicated in precipitating complications, including pulmonary infections, heart failure, and arrhythmias. Management of these complications is challenging and is associated with a poor prognosis, leading to a significant reduction in both the quality of life and life expectancy of patients with the condition (2). As a significant contributor to cardiovascular disease-related disability and death, the incidence and prevalence of DMVD are on the rise. This trend is particularly pronounced in certain developed countries, where DMVD has surpassed rheumatic mitral valve disease (3, 4). Consequently, both developed and developing countries face considerable challenges to their cardiovascular healthcare systems as the population ages.

The 2019 Global Burden of Diseases (GBD) study was utilized by Wang et al. and Liu et al. to report a significant increase in the burden of DMVD (5, 6). The incidence cases of DMVD were estimated at 1.064 million cases, while the disease prevalence cases were notably up to 24.229 million in 2019. The death cases attributed to DMVD accounted for 340,000. Additionally, the disability-adjusted life years (DALYs) for DMVD were calculated at 883,000 in 2019. The economic strain of DMVD impacts individuals and families, places significant demands on healthcare systems, and affects national economies (7). However, prior research has been limited in its comprehensive analysis across subgroups defined by age, year, and socio-demographic index (SDI), and it has not adequately addressed long-term projections of the disease's future burden. Furthermore, these studies have not sufficiently explored the inequality in disease burden cross-countries.

The GBD study provides a solid framework to assess trends and projection in DMVD incidence, prevalence, deaths, and DALYs. This research utilizes information from the 2021 GBD study to investigate trends in disease burden from 1990 to 2021, (8) with forecasts extending to 2050, thereby offering a comprehensive insight into the DMVD burden across various nations, regions, and global. Our study aims to improve understanding of the complex epidemiology of DMVD and inform the development of targeted strategies and policies aimed at mitigating its impact.

## Methods

### Data source

Utilizing the latest epidemiological data and refined standardized methods, the 2021 GBD study presents a comprehensive evaluation of health impairments linked to 369 diseases, injuries, and disabilities, alongside 88 risk factors, spanning 204 countries and territories (9). Although the study spans from 1980 to the present, considering the historical significance, policy impact, data availability, and methodological development of 1990, it is commonly used as the baseline year for comparison with the present (10). Previous studies have provided a comprehensive introduction to the design and methodology of the GBD information (11).

### Data collection

DMVD is categorized within the 2021 GBD study cause list, aligning with the International Classification of Diseases (ICD) coding system, where it corresponds to codes 424.0 under ICD-9 and I34.0 under ICD-10. The 1990–2021 dataset was extracted from the 2021 GBD study, which segments the age spectrum into 20 increments of 5 years, ranging from 0 to 100 years. In terms of locations, our analysis encompasses not only the aggregated global data and the specific data for 204 countries and territories but also incorporates data for 53 distinct GBD regions (9).

SDI is a composite measure reflecting a nation's or region's level of development, quantified by average income per capita, educational attainment among individuals aged 15 years and above, and total fertility rates for those under 25 years. It scales from 0 to 1, with higher values indicating a more advanced socioeconomic standing. Utilizing the SDI values from 2021, countries and territories are categorized into five distinct groups: high SDI, high-middle SDI, middle SDI, low-middle SDI, and low SDI. These indicators are also selected for analysis in terms of locations (9).

### Statistical analysis

After extracting variables such as age, time, and location, the cases and age-standardized rate (ASR) per 100,000 population of incidence, prevalence, death, and DALYs with 95% uncertainty interval (UI) obtained from the GBD dataset were utilized to illustrate the current state of DMVD burden in 2021. DALYs represent the sum of years of life lost due to premature death and years lived with disability. Temporal patterns were evaluated utilizing the estimated annual percentage changes (EAPC), accompanied by 95% confidence intervals (CI), which were ascertained through linear regression (12). The model employed was Y = *α* + *β*X + *ε*, where Y signifies the natural logarithm of the ASR, X denotes the calendar years, *α* represents the baseline value of log(ASR) at year zero, *β* quantifies the annual change in log(ASR), and *ε* captures the residual error. The EAPC was computed as 100 × [exp(*β*) − 1]. A positive EAPC value, along with a 95% CI above zero, infers an upward trend in ASR, while a negative value suggests a downward trend.

The exponential smoothing (ES) and autoregressive integrated moving average (ARIMA) models have been strategically applied to forecast the trajectory of DMVD (13). For the ES model, we employed the Holt-Winters additive method to account for both trend and seasonal components in the time-series data. Smoothing parameters were optimized using the maximum likelihood estimation. For ARIMA, series stationarity was assessed using the Augmented Dickey-Fuller test, and appropriate differencing (*d* = 1) was applied. Autoregressive and moving average orders were determined through autocorrelation function and partial autocorrelation function plots to predict future disease patterns.

To evaluate the relationship between burden of DMVD and socio-demographic development, frontier analysis was utilized to ascertain the minimum potentially achievable burden of disease, based on a country's development status as measured by the SDI (14). This analysis employed non-parametric data envelope analysis to evaluate the minimum ASR for conditions across various SDI levels. The frontier line indicated those countries and territories exhibiting the lowest ASR in relation to their respective SDI values. The gap between the actual observed ASR of a country and the ASR that could feasibly be achieved is referred to as the distance from the frontier line, known as the effective difference. This effective difference has the potential to be diminished or entirely removed, contingent on the socio-demographic resources of the country. By comparing observed health outcomes with the best performance observed within similar development contexts, frontier analysis reveals the unrealized health gain that could be achieved if all countries or territories were to perform at the level of the best-performing peers within their SDI category.

To assess disparities in the age-standardized DALY rate (ASDALYR) for DMVD globally and across 204 countries, we employed the slope inequality index and the concentration index (15). The slope inequality index quantifies absolute inequality by estimating the difference in ASDALYR between the most and least advantaged subgroups through a weighted least-squares regression of health outcomes on socioeconomic rank. Conversely, the concentration index gauges relative inequality of disease burden, revealing the degree among the disadvantaged or advantaged groups. It is calculated as twice the area between the concentration curve and the line of equality, with values ranging from −1 to +1. These metrics offer a standardized lens through which to view health inequities.

All analytical procedures and graphical representations were performed using the R statistical software (version 4.2.3) and World Health Organization's Health Equity Assessment Toolkit.

## Results

### Burden of DMVD in 2021

The age-standardized incidence rate (ASIR) of DMVD for the combined sexes in 2021 was measured at 12.98 per 100,000 (95% UI, 12.11–13.89), with males at 17.67 (16.48–18.99) per 100,000 and females at 8.66 (8.09–9.25) per 100,000 (Table 1). In terms of the age-standardized prevalence rate (ASPR) of DMVD for both sexes together, it was documented as 182.13 per 100,000 (169.85–196.07), with males at 257.92 (240.96–278.21) per 100,000 and females at 121.11 (112.92–129.82) per 100,000. The age-standardized death rate (ASDR) of DMVD for both genders combined was reported at 0.46 per 100,000 (0.39–0.51), with males at 0.41 (0.36–0.46) per 100,000 and females at 0.49 (0.40–0.57) per 100,000. Finally, the ASDALYR for both sexes together stood at 11.36 per 100,000 (9.87–13.61), with males at 11.57 (9.66–14.07) per 100,000 and females at 11.28 (9.16–13.64) per 100,000 (Supplementary Figure S1).

**Table 1:** The case number and ASR of DMVD in incidence, prevalence, deaths, and DALYs in 1990 and 2021.

Year 2021	Number of cases (95% UI)				The age-standardized rate/1,00,000 (95% UI)			
	Incidence	Prevalence	Deaths	DALYs	Incidence	Prevalence	Deaths	DALYs
Global	1,162,558 (1,084,358–1,244,874)	15,494,647 (14,457,324–16,702,738)	36,844 (31,883–41,572)	943,258 (818,239–1,134,001)	12.98 (12.11–13.89)	182.13 (169.95–196.07)	0.46 (0.39–0.51)	11.36 (9.87–13.61)
Sex								
Female	405,244 (378,721–433,089)	5,665,310 (5,281,762–6,073,695)	22,675 (18,541–2,6629)	509,344 (414,981–614,453)	8.66 (8.09–9.25)	121.11 (112.92–129.82)	0.49 (0.4–0.57)	11.28 (9.16–13.64)
Male	757,313 (705,850–813,961)	9,829,338 (9,179,556–10,61,5,234)	14,168 (12,368–16,111)	433,914 (358,440–527,231)	17.67 (16.48–18.99)	257.92 (240.96–278.21)	0.41 (0.36–0.46)	11.57 (9.66–14.07)
Age								
15–19 years	600 (470–747)	946 (747–1,193)	260 (191–330)	18,865 (13,875–23,939)	0.1 (0.08–0.12)	0.15 (0.12–0.19)	0.04 (0.03–0.05)	3.02 (2.22–3.84)
20–24 years	1,774 (1,398–2,203)	6,148 (4,869–7,716)	348 (265–440)	23,515 (17,901–29,799)	0.3 (0.23–0.37)	1.03 (0.82–1.29)	0.06 (0.04–0.07)	3.94 (3–4.99)
25–29 years	3,023 (2,397–3,746)	16,615 (13,238–20,722)	376 (288–452)	23,574 (18,096–28,333)	0.51 (0.41–0.64)	2.82 (2.25–3.52)	0.06 (0.05–0.08)	4.01 (3.08–4.82)
30–34 years	4,422 (3,520–5,463)	33,601 (26,886–41,719)	447 (359–542)	25,867 (20,742–31,356)	0.73 (0.58–0.9)	5.56 (4.45–6.9)	0.07 (0.06–0.09)	4.28 (3.43–5.19)
35–39 years	5,494 (4,391–6,772)	53,797 (43,257–66,509)	559 (449–677)	29,594 (23,770–35,824)	0.98 (0.78–1.21)	9.59 (7.71–11.86)	0.1 (0.08–0.12)	5.28 (4.24–6.39)
40–44 years	8,541 (6,608–10,927)	79,713 (66,832–95,454)	739 (610–882)	35,528 (29,356–42,283)	1.71 (1.32–2.18)	15.93 (13.36–19.08)	0.15 (0.12–0.18)	7.1 (5.87–8.45)
45–49 years	13,310 (7,936–20,516)	125,878 (102,581–151,859)	870 (732–1,061)	37,714 (31,809–45,887)	2.81 (1.68–4.33)	26.58 (21.66–32.07)	0.18 (0.15–0.22)	7.96 (6.72–9.69)
50–54 years	46,988 (38,706–58,638)	238,379 (194,941–300,372)	1,151 (959–1,368)	44,863 (37,355–53,086)	10.56 (8.7–13.18)	53.58 (43.81–67.51)	0.26 (0.22–0.31)	10.08 (8.4–11.93)
55–59 years	104,147 (82,188–130,813)	582,819 (494,775–703,864)	1,705 (1,423–2,028)	60,003 (50,320–70,755)	26.32 (20.77–33.06)	147.28 (125.03–177.87)	0.43 (0.36–0.51)	15.16 (12.72–17.88)
60–64 years	224,643 (206,005–246,615)	1,231,968 (1,066,354–1,456,093)	2,188 (1,924–2,532)	71,181 (62,087–82,773)	70.19 (64.37–77.06)	384.93 (333.19–454.96)	0.68 (0.6–0.79)	22.24 (19.4–25.86)
65–69 years	381,421 (350,098–416,643)	2,501,089 (2,316,673–2,740,086)	2,876 (2,450–3,318)	90,964 (78,014–111,071)	138.27 (126.92–151.04)	906.71 (839.85–993.35)	1.04 (0.89–1.2)	32.98 (28.28–40.27)
70–74 years	287,466 (262,667–316,624)	3,633,715 (3,393,547–3,902,759)	3,539 (3,059–4,067)	110,288 (90,964–140,688)	139.66 (127.61–153.82)	1,765.32 (1,648.64–1,896.02)	1.72 (1.49–1.98)	53.58 (44.19–68.35)
75–79 years	69,302 (62,620–76,784)	2,973,841 (2,759,872–3,196,224)	4,147 (3,599–4,763)	108,273 (86,781–138,302)	52.55 (47.48–58.22)	2,254.88 (2,092.64–2,423.5)	3.14 (2.73–3.61)	82.1 (65.8–104.87)
80–84 years	7,165 (3,619–11,808)	2,192,416 (2,036,952–2,362,417)	5,008 (4,228–5,651)	102,144 (84,020–130,415)	8.18 (4.13–13.48)	2,503.24 (2,325.74–2,697.34)	5.72 (4.83–6.45)	116.63 (95.93–148.9)
85–89 years	3,092 (1,758–4,869)	1,192,625 (1,108,730–1,285,467)	5,586 (4,458–6,313)	81,959 (67,488–101,412)	6.76 (3.84–10.65)	2,608.44 (2,424.95–2,811.5)	12.22 (9.75–13.81)	179.26 (147.61–221.8)
90–94 years	954 (532–1,576)	485,250 (448,558–523,881)	4,622 (3,489–5,239)	52,998 (42,376–62,015)	5.34 (2.97–8.81)	2,712.51 (2,507.4–2,928.45)	25.84 (19.5–29.28)	296.25 (236.88–346.66)
95+ years	216 (90–448)	145,847 (134,542–157,295)	2,422 (1,690–2,820)	25,928 (19,890–30,411)	3.96 (1.65–8.22)	2,675.94 (2,468.52–2,885.98)	44.44 (31.01–51.73)	475.71 (364.94–557.98)
SDI region								
High SDI	569,743 (532,337–610,609)	8,149,325 (7,632,524–8,750,587)	16,264 (13,478–17,843)	334,695 (286,438–404,530)	28.49 (26.66–30.52)	364.24 (341.57–390.64)	0.65 (0.55–0.7)	15.01 (13.02–17.98)
High-middle SDI	333,052 (311,758–356,305)	4,486,461 (4,187,532–4,821,465)	6,887 (6,041–7,711)	184,649 (156,704–228,858)	16.01 (14.99–17.11)	223.55 (208.8–240.01)	0.37 (0.32–0.41)	9.77 (8.33–12.01)
Middle SDI	191,670 (178,708–207,179)	2,131,567 (1,978,434–2,317,641)	5,692 (4,775–7,097)	175,874 (152,861–212,348)	6.75 (6.29–7.29)	83.14 (77.41–89.93)	0.23 (0.19–0.29)	6.76 (5.85–8.19)
Low-middle SDI	55,269 (49,821–61,569)	597,241 (530,959–673,732)	5,465 (4,214–6,975)	165,113 (129,856–204,532)	3.68 (3.32–4.09)	44.67 (39.91–50.05)	0.4 (0.31–0.53)	10.62 (8.41–13.21)
Low SDI	11,811 (10,737–13,005)	115,513 (101,726–130,949)	2,479 (1,698–3,446)	81,577 (56,425–109,180)	2.16 (1.97–2.37)	24.77 (21.96–27.95)	0.51 (0.34–0.73)	12.8 (9–17.54)

Year 1990	Number of cases (95% UI)				The age-standardized rate/100,000 (95% UI)			
	Incidence	Prevalence	Deaths	DALYs	Incidence	Prevalence	Deaths	DALYs
Global	566,261 (523,330–609,607)	7,111,757 (6,572,737–7,719,089)	23,954 (21,032–26,296)	645,877 (553,701–749,210)	13.73 (12.69–14.76)	193.04 (178.74–208.37)	0.71 (0.63–0.78)	16.79 (14.68–19.28)
Sex								
Female	206,854 (191,173–222,317)	2,787,243 (2,574,278–3,004,423)	15,396 (13,094–17,330)	374,581 (312,335–443,051)	9.48 (8.76–10.2)	134.53 (124.14–144.82)	0.79 (0.67–0.89)	17.62 (14.91–20.61)
Male	359,407 (332,181–388,636)	4,324,515 (3,989,733–4,706,437)	8,558 (7,513–9,479)	271,296 (230,849–319,851)	18.48 (17.07–19.99)	272.36 (252.43–294.91)	0.59 (0.51–0.64)	15.87 (13.32–18.94)
Age								
15–19 years	591 (468–742)	951 (745–1,188)	263 (172–354)	19,108 (12,509–25,682)	0.11 (0.09–0.14)	0.18 (0.14–0.23)	0.05 (0.03–0.07)	3.68 (2.41–4.94)
20–24 years	1,720 (1,364–2,153)	6,136 (4,824–7,659)	326 (210–433)	22,081 (14,198–29,287)	0.35 (0.28–0.44)	1.25 (0.98–1.56)	0.07 (0.04–0.09)	4.49 (2.89–5.95)
25–29 years	2,830 (2,245–3,526)	16,098 (12,779–20,021)	352 (249–460)	22,096 (15,625–28,842)	0.64 (0.51–0.8)	3.64 (2.89–4.52)	0.08 (0.06–0.1)	4.99 (3.53–6.52)
30–34 years	3,761 (2,984–4,658)	29,589 (23,571–36,603)	375 (280–471)	21,682 (16,198–27,203)	0.98 (0.77–1.21)	7.68 (6.12–9.5)	0.1 (0.07–0.12)	5.63 (4.2–7.06)
35–39 years	4,435 (3,528–5,473)	44,901 (35,751–55,265)	456 (349–565)	24,120 (18,501–29,855)	1.26 (1–1.55)	12.75 (10.15–15.69)	0.13 (0.1–0.16)	6.85 (5.25–8.48)
40–44 years	6,662 (5,143–8,616)	62,227 (51,772–75,212)	542 (421–656)	26,082 (20,224–31,465)	2.33 (1.8–3.01)	21.72 (18.07–26.25)	0.19 (0.15–0.23)	9.1 (7.06–10.98)
45–49 years	9,215 (5,505–14,185)	84,228 (67,985–102,439)	616 (490–742)	26,714 (21,293–32,059)	3.97 (2.37–6.11)	36.27 (29.28–44.12)	0.27 (0.21–0.32)	11.51 (9.17–13.81)
50–54 years	31,898 (25,695–40,510)	163,415 (132,785–207,126)	870 (711–1,030)	33,893 (27,706–39,707)	15.01 (12.09–19.06)	76.88 (62.47–97.44)	0.41 (0.33–0.48)	15.94 (13.03–18.68)
55–59 years	65,260 (50,091–83,861)	371,597 (309,952–456,847)	1,293 (1,084–1,486)	45,332 (38,306–51,884)	35.24 (27.05–45.28)	200.65 (167.36–246.68)	0.7 (0.59–0.8)	24.48 (20.68–28.02)
60–64 years	123,473 (110,869–137,987)	769,186 (652,641–927,362)	1,821 (1,584–2,029)	58,111 (50,733–66,135)	76.88 (69.03–85.91)	478.92 (406.35–577.4)	1.13 (0.99–1.26)	36.18 (31.59–41.18)
65–69 years	171,861 (154,371–192,694)	1,265,714 (1,151,587–1,409,590)	2,452 (2,155–2,695)	71,590 (62,370–83,866)	139.04 (124.89–155.89)	1,023.96 (931.63–1,140.36)	1.98 (1.74–2.18)	57.92 (50.46–67.85)
70–74 years	108,937 (96,079–123,700)	1,473,057 (1,361,219–1,590,256)	2,705 (2,394–2,955)	72,482 (62,508–86,925)	128.67 (113.49–146.11)	1,739.94 (1,607.84–1,878.38)	3.2 (2.83–3.49)	85.61 (73.83–102.67)
75–79 years	31,649 (27,679–36,160)	1,440,404 (1,325,751–1,566,720)	3,736 (3,374–4,026)	82,877 (71,225–100,404)	51.42 (44.97–58.74)	2,340.01 (2,153.75–2,545.21)	6.07 (5.48–6.54)	134.64 (115.71–163.11)
80–84 years	2,749 (1,345–4,600)	873,294 (801,732–952,301)	3,567 (3,125–3,898)	62,634 (53,709–76,007)	7.77 (3.8–13)	2,468.61 (2,266.32–2,691.95)	10.08 (8.83–11.02)	177.05 (151.82–214.86)
85–89 years	963 (522–1,572)	377,009 (345,934–412,645)	2,734 (2,295–3,018)	36,772 (31,162–43,524)	6.37 (3.46–10.4)	2,494.92 (2,289.27–2,730.75)	18.09 (15.19–19.97)	243.35 (206.22–288.03)
90–94 years	217 (119–358)	108,205 (98,678–118,154)	1,381 (1,107–1,568)	15,243 (12,688–17,690)	5.07 (2.77–8.36)	2,525.1 (2,302.78–2,757.26)	32.23 (25.83–36.59)	355.71 (296.1–412.81)
95+ years	39 (17–81)	25,747 (23,540–28,209)	465 (346–531)	5,059 (4,031–5,878)	3.86 (1.67–7.99)	2,528.98 (2,312.14–2,770.76)	45.66 (33.97–52.19)	496.91 (395.91–577.35)
SDI region								
High SDI	289,042 (268,397–311,012)	3,870,206 (3,585,779–4,185,413)	13,007 (11,846–13,785)	286,597 (261,114–323,954)	26.2 (24.31–28.18)	337.89 (313.22–365.83)	1.18 (1.07–1.25)	25.98 (23.76–29.18)
High-middle SDI	180,837 (166,880–195,319)	2,248,064 (2,073,439–2,447,323)	4,443 (3,962–4,833)	,134,087 (115,261–158,668)	17.16 (15.85–18.51)	235.91 (218.2–256.06)	0.51 (0.45–0.56)	14.09 (12.16–16.73)
Middle SDI	67,453 (61,686–73,335)	699,387 (632,990–774,419)	2,706 (2,210–3,196)	97,118 (81,912–113,664)	6.21 (5.7–6.74)	77.36 (70.34–85.01)	0.28 (0.23–0.34)	8.33 (6.99–9.75)
Low-middle SDI	22,978 (20,516–25,650)	236,184 (206,976–267,733)	2,430 (1,707–3,236)	82,288 (57,207–112,961)	3.61 (3.22–4.05)	44.25 (38.82–49.94)	0.41 (0.29–0.55)	11.37 (8.18–15.04)
Low SDI	5,294 (4,731–5,856)	49,418 (42,745–56,717)	1,336 (776–1,875)	44,878 (23,753–63,997)	2.18 (1.97–2.42)	24.13 (21.03–27.41)	0.62 (0.38–0.91)	16.17 (9.41–22.69)

ASR, age-standardized rate; DMVD, degenerative mitral valve disease; DALYs, disability-adjusted life-years; SDI, socio-demographic index; UI, uncertainty interval.

**Figure 1:**
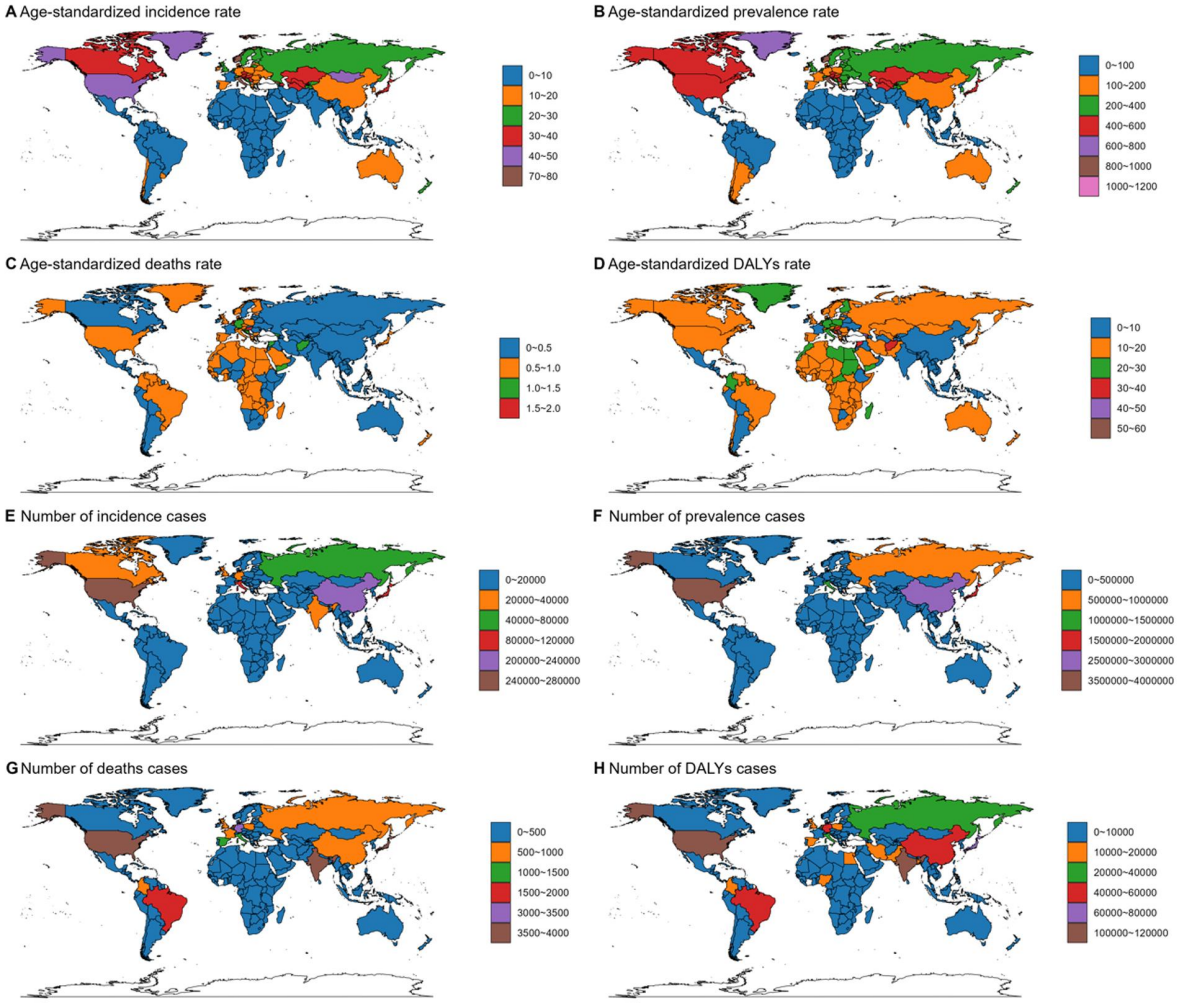
Age-standardized rate and number of cases of incidence **(A,E)**, prevalence **(B,F)**, deaths **(C,G)**, and DALYs **(D,H)** of DMVD in both sexes combined in 204 countries around the world in 2021. DALYs, disability-adjusted life years; DMVD, degenerative mitral valve disease. World maps generated using the ggmap package (https://cran.r-project.org/web/packages/ggmap/index.html), licensed under GPLv2.

In 2021, high-SDI quintiles were highest in terms of ASIR, ASPR, ASDR, and ASDALYR for DMVD (Supplementary Figure S2, Table 1). Similarly, North America and high-income North America recorded the highest ASIR and ASPR, while central Europe showed the highest ASDR and ASDALYR (Supplementary Figure S3, Supplementary Table S1). Notably, as shown in Figure 1 and Supplementary Table S1, Georgia reported the highest ASIR and ASPR, whereas Serbia had the highest ASDR and ASDALYR. In terms of the number of cases, the four indicators demonstrate comparable outcomes. As presented in Supplementary Figures S4–S5, the correlation analysis indicates that the four epidemiological indicators are stable across countries with varying levels of SDI. ASIR and ASPR gradually increase with rising SDI, and the rate of increase accelerates progressively. However, ASDR and ASDALYR exhibit an M-shaped pattern, with increases and decreases observed on either side of SDI values of 0.25 and 0.85, respectively.

As illustrated in Supplementary Figure S6 and Table 1, the incidence number reached its peak among individuals aged 65–69 years, reaching 381,421 (350,098–416,643). While the prevalence and DALYs peaked in the 70–74 years group, with 3,633,715 (3,393,547–3,902,759) and 110,288 (90,964–140,688) respectively. The death count was highest for those aged at 85–89 years, at 5,586 (4,458–6,313). The age group with the highest ASIR was also 70–74 years, reaching 139.66 (127.61–153.82). ASPR reached a maximum at 90–94 years, with a count of 2,712.51 (2,507.4–2,928.45). Meanwhile, ASDALYR and ASDR were highest in the 95 + age, at 475.71 (364.94–557.98) and 44.44 (31.01–51.73) respectively.

### The trend of DMVD burden from 1990 to 2021

As depicted in Supplementary Figure S7 and Table 1, from 1990 to 2021, the incidence cases of DMVD rose from 566,261 (95% UI, 523,330–609,607) to 1,162,558 (1,084,358–1,244,874), representing an increase of 105.3% (77.9–137.9). Concurrently, the prevalence cases increased from 7,111,757 (6,572,737–7,719,089) to 15,494,647 (14,457,324–16,702,738), marking a rise of 117.9% (87.3–154.1). Furthermore, the death cases attributed DMVD increased by 53.8% (21.2–97.7), moving from 23,954 (21,032–26,296) to 36,844 (31,883–41,572). Meanwhile, the DALYs increased from 645,877 (553,701–749,210) to 943,258 (818,239–1,134,001), an increase of 46.0% (9.2–104.8). A comparable trend is observed between males and females (Supplementary Figure S8).

**Figure 2:**
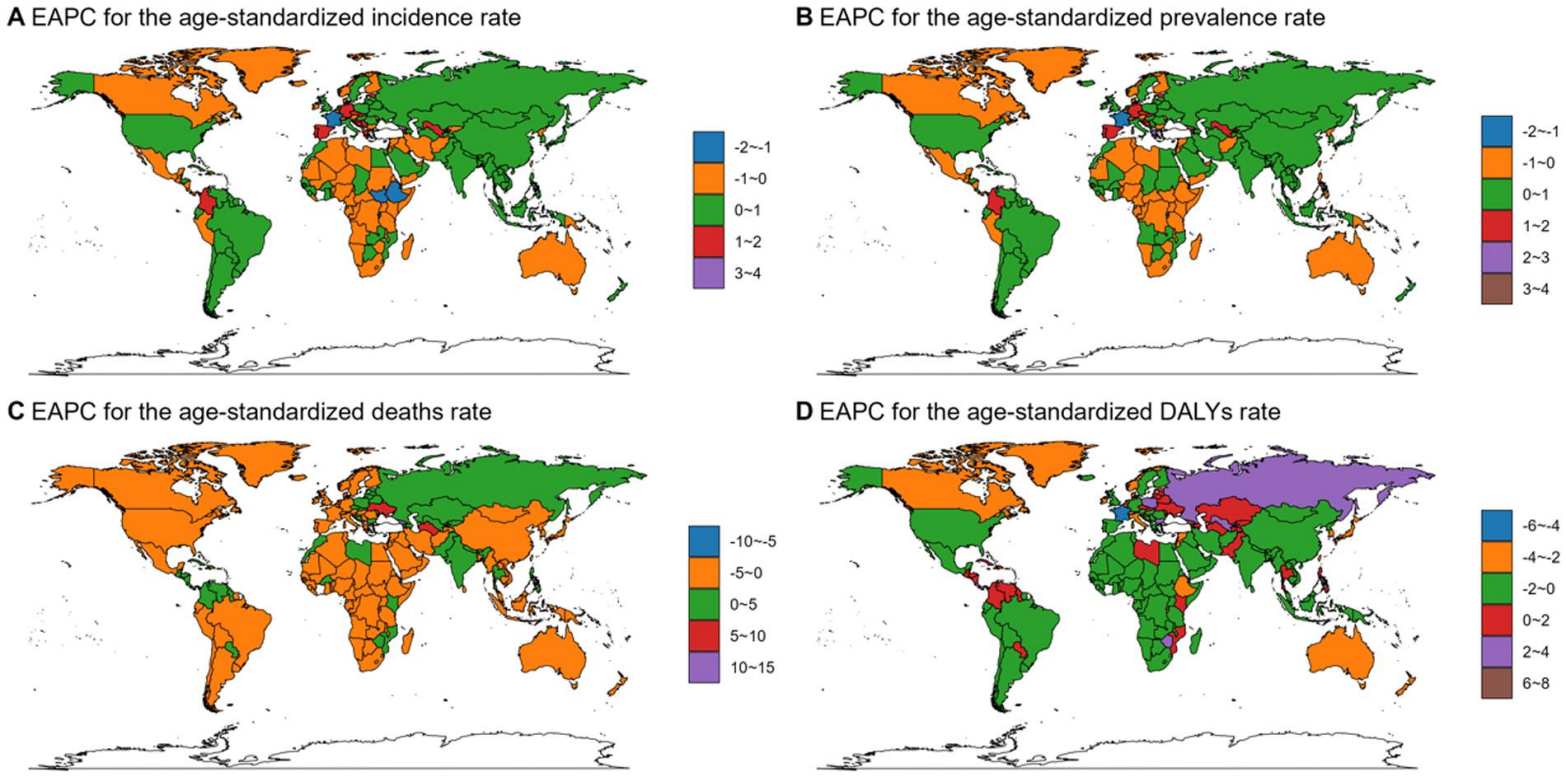
EAPC for the age-standardized rate of incidence **(A)**, prevalence **(B)**, deaths **(C)**, and DALYs **(D)** for DMVD in different countries and regions from 1990 to 2021. EAPC, estimated annual percentage change; DALYs, disability-adjusted life years; DMVD, degenerative mitral valve disease. World maps generated using the ggmap package (https://cran.r-project.org/web/packages/ggmap/index.html), licensed under GPLv2.

The temporal trends of ASIR, ASPR, ASDR and ASDALYR across diverse regions and countries from 1990 to 2021, stratified by age and sex groups, are comprehensively presented in Figure 2, Supplementary Figures S7–S9, Table 2 and Supplementary Table S1. During the same period, the global ASIR for DMVD displayed a stable trend [EAPC, −0.02 (95% CI, −0.07–0.02)]. However, the ASIR for DMVD remained constant in males [EAPC, 0 (−0.05–0.04)] while exhibiting a decrease in females [EAPC, −0.11 (–0.16–−0.06)]. Additionally, middle SDI quintiles [EAPC, 0.47 (0.38–0.56)], along with Central Asia [EAPC, 1.13 (0.96–1.29)], demonstrated the most significant increasing trend in ASIR. At the national level, Georgia [EAPC, 3.63 (3.01–4.25)] showed the most significant increasing trend in ASIR for DMVD.

**Table 2:** EAPC in incidence, prevalence, deaths, and DALYs rate between 1990 and 2021.

Groups	EAPC of incidence rate, % (95% CI)	EAPC of prevalence rate, % (95% CI)	EAPC of deaths rate, % (95% CI)	EAPC of DAlYs rate, % (95% CI)
Global	−0.02 (−0.07–0.02)	−0.1 (−0.13−0.06)	−1.49 (−1.6–1.38)	−1.32 (−1.41–1.22)
Sex				
Female	−0.11 (−0.16–0.06)	−0.24 (−0.29–0.2)	−1.61 (−1.71–1.51)	−1.51 (−1.61–1.4)
Male	0 (−0.05–0.04)	−0.09 (−0.13–0.06)	−1.24 (−1.37–1.11)	−1.07 (−1.15–0.98)
Age				
15–19 years	−0.63 (−0.75–0.52)	−0.69 (−0.81–0.57)	−0.81 (−0.91–0.7)	−0.81 (−0.92–0.7)
20–24 years	−0.61 (−0.69–0.53)	−0.7 (−0.79–0.61)	−0.71 (−0.85–0.56)	−0.71 (−0.85–0.56)
25–29 years	−0.6 (−0.71–0.49)	−0.71 (−0.83–0.58)	−0.76 (−0.86–0.65)	−0.76 (−0.87–0.65)
30–34 years	−0.81 (−1.03–0.6)	−0.93 (−1.16–0.71)	−0.86 (−0.93–0.78)	−0.86 (−0.93–0.78)
35–39 years	−0.88 (−1.09–0.66)	−0.99 (−1.22–0.77)	−0.91 (−1.07–0.76)	−0.92 (−1.07–0.76)
40–44 years	−1.24 (−1.41–1.07)	−1.14 (−1.29–0.98)	−1.04 (−1.25–0.84)	−1.05 (−1.25–0.84)
45–49 years	−1.5 (−1.65–1.34)	−1.26 (−1.39–1.13)	−1.41 (−1.56–1.27)	−1.42 (−1.56–1.27)
50–54 years	−1.14 (−1.19–1.09)	−1.27 (−1.32–1.21)	−1.58 (−1.71–1.45)	−1.58 (−1.71–1.46)
55–59 years	−0.82 (−0.9–0.75)	−0.94 (−1.03–0.86)	−1.59 (−1.8–1.39)	−1.58 (−1.77–1.38)
60–64 years	−0.09 (−0.17–0.01)	−0.65 (−0.72–0.58)	−1.83 (−2.02–1.63)	−1.74 (−1.92–1.56)
65–69 years	0.26 (0.17–0.35)	−0.23 (−0.29–0.17)	−2.15 (−2.34–1.96)	−1.85 (−2–1.69)
70–74 years	0.32 (0.26–0.39)	0.05 (0–0.1)	−2.26 (−2.46–2.07)	−1.7 (−1.84–1.56)
75–79 years	0.21 (0.11–0.3)	0.05 (−0.04–0.14)	−2.22 (−2.37–2.07)	−1.6 (−1.7–1.5)
80–84 years	0.09 (0.04–0.15)	0.1 (0.01–0.2)	−1.92 (−2.03–1.81)	−1.38 (−1.45–1.32)
85–89 years	0.11 (0.03–0.18)	0.3 (0.18–0.43)	−1.17 (−1.24–1.1)	−0.87 (−0.94–0.81)
90–94 years	0.08 (0–0.15)	0.42 (0.33–0.51)	−0.58 (−0.68–0.48)	−0.44 (−0.53–0.36)
95+ years	0.01 (−0.11–0.12)	0.25 (0.19–0.3)	−0.08 (−0.22–0.06)	−0.13 (−0.24–0.02)
SDI region				
High SDI	0.41 (0.34–0.48)	0.31 (0.25–0.36)	−2.06 (−2.2–1.91)	−1.87 (−2–1.74)
High-middle SDI	−0.06 (−0.13–0)	−0.02 (−0.07–0.04)	−1.13 (−1.22–1.03)	−1.24 (−1.32–1.17)
Middle SDI	0.47 (0.38–0.56)	0.4 (0.32–0.48)	−0.61 (−0.74–0.49)	−0.65 (−0.75–0.55)
Low-middle SDI	0.07 (0.02–0.11)	0.07 (0.03–0.12)	−0.04 (−0.12–0.03)	−0.21 (−0.26–0.16)
Low SDI	0.01 (−0.02–0.05)	0.14 (0.1–0.18)	−0.69 (−0.82–0.56)	−0.88 (−0.99–0.77)

EAPC, estimated annual percentage change; DALYs, disability-adjusted life years; SDI, socio-demographic index.

Between 1990 and 2021, the ASPR of DMVD experienced a steady trend [EAPC, −0.1 (95% CI, −0.13–0.06)]. Both males [EAPC, −0.09 (–0.13–0.06)] and females [−0.24 (−0.29–0.2)] showed a reduction. Moreover, the ASPR increased in all SDI quintile except high-middle SDI, with notable increases observed in Central Asia [EAPC, 1.25 (1.06–1.45)] and Georgia [EAPC, 3.53 (2.94–4.12)].

A declining trend in ASDR was noted during the study period [EAPC, −1.49 (−1.6–−1.38)], with females showing a decrease [EAPC, −1.61 (−1.71–−1.51)] and males exhibiting a slightly lesser decline [EAPC, −1.24 (−1.37–−1.11)]. All SDI quintiles, excluding low-middle, exhibit a significant decreasing trend, with the high SDI quintiles experiencing the most notable decline of −2.06 (−2.2–−1.91) for EAPC. As for the GBD region, Central Asia demonstrates the most pronounced increase [EAPC, 5.52 (4.86–6.19)], while East Asia shows the most significant decrease of −3.5 (−4.03–−2.97) for EAPC.

Similarly, the ASDALYR showed a decreasing trend [EAPC, −1.32 (95% CI, −1.41–−1.22)]. Specifically, it decreased for males [EAPC, −1.07 (−1.15–−0.98)] and for females [EAPC, −1.51 (−1.61–−1.4)]. All SDI quintiles experienced reductions, with the high-SDI quintiles showing the most significant decline [EAPC, −1.81 (−2–−10-.74)]. Additionally, at the GBD region level, Central Asia experienced the fastest growth [EAPC 2.62 (2.25–2.99)], while High-income Asia Pacific saw the most significant decline [EAPC −2.76 (−3.13–−2.39)]. Furthermore, at the national levels, Georgia [EAPC, 7.52 (6.85–8.21)] is facing the most increasing in ASDALYR related to DMVD, while Lebanon reported the most significant decrease of −4.59 (−4.88–4.3) for EAPC.

As indicated in Table 2 and Supplementary Figure S10, between 1990 and 2021, The overall trend for the number of cases across the four indicators in different age groups is increasing annually, but the ASR shows a different pattern. The ASIR of DMVD, divided into 5-year age groups, showed an upward trend among individuals aged 65 years and older, a stable pattern for those who are 95 + years, and a downward trend among those under 65. The most significant rise was noted in the 70–74 age group [EAPC, 0.32 (0.26–0.39)], whereas the largest decline was seen in the 45–49 years group [EAPC, −1.7 (−1.8–−1.5)]. Additionally, the ASPR of DMVD revealed an increasing tendency in individuals aged ≥80 years, remained stable for those aged 70–79, and exhibited a decreasing trend for individuals younger than 70. The 90–94 age category experienced the most marked increase [EAPC, 0.42 (0.33–0.51)], in contrast, the most significant decrease was reported in the 50–54 years group [EAPC, −1.27 (−1.32–−1.21)]. ASDR and ASDALYR attributable to DMVD showed a declining trend across nearly all age categories, except for a stable ASDR for the 95 + age group. Within the 70–74 age range, the ASDR witnessed the sharpest decline [EAPC, −2.26 (−2.46–−2.07)], while the 65–69 years group displayed the greatest reduction in ASDALYR [EAPC, −1.85 (−2–−1.69)].

### Prediction of DMVD epidemic

**Figure 3:**
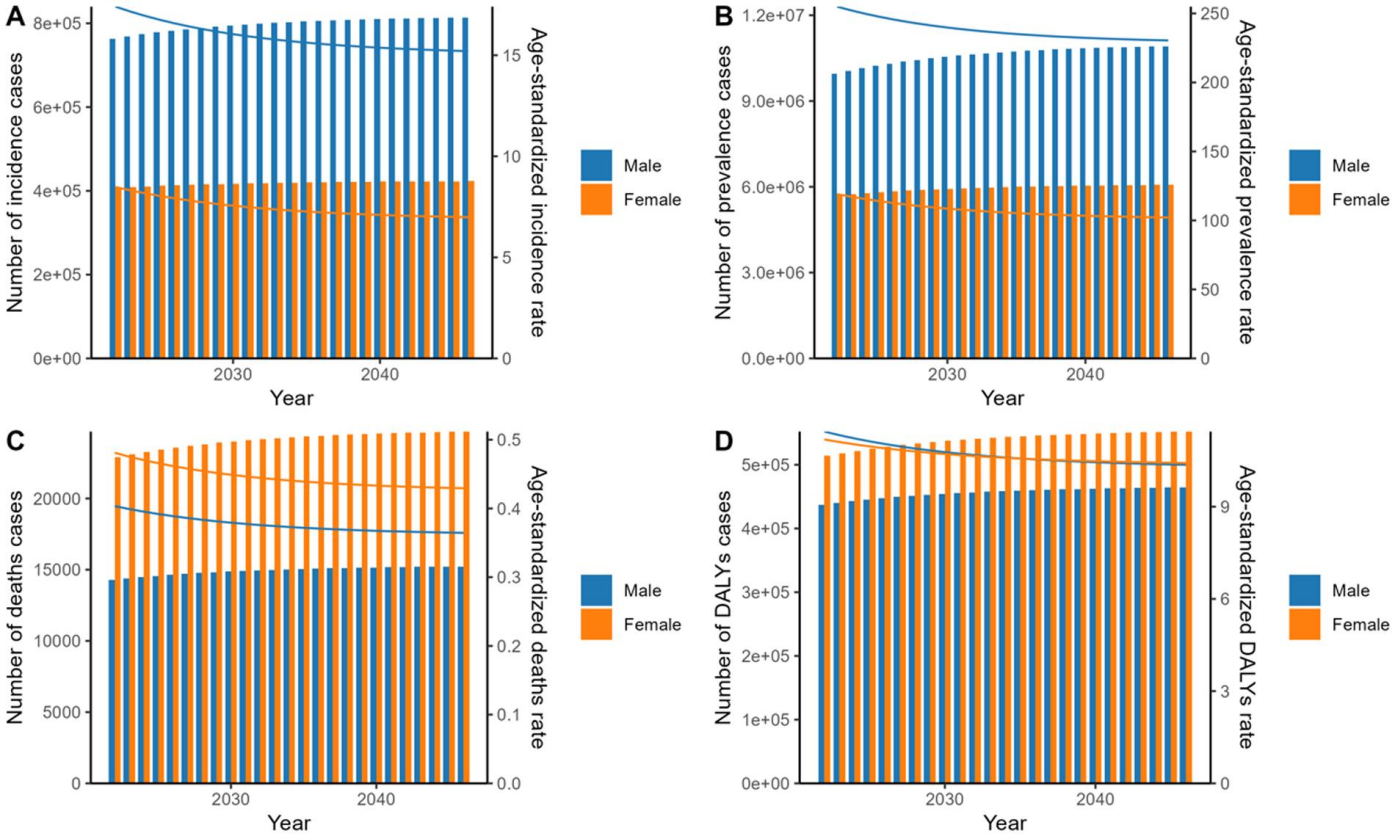
Prediction of age-standardized rate and number of cases of incidence **(A)**, prevalence **(B)**, deaths **(C)**, and DALYs **(D)** by sex using ES model for 2022–2050. DALYs, disability-adjusted life years; ES, exponential smoothing.

The ES model was used to predict trends until 2050. As depicted in Figure 3 and Supplementary Table S2. The number of incidence, prevalence, death, and DALYs are expected to show a moderate upward trend from 2022 to 2050. The estimated incidence is expected to rise from 1,170,601 in 2022 to 1,239,195 in 2050. Similarly, the estimated prevalence is projected to climb from 15,655,462 in 2022 to 17,026,986 in 2050. Death is also anticipated to increase, from an estimated 37,179 in 2022 to 40,007 in 2050. Furthermore, the estimated number of DALYs is expected to grow from 951,277 in 2022 to 1,018,540 in 2050. However, the ASIR, ASPR, ASDR, and ASDALYR are projected to gradually decrease, with the rate of decrease expected to slow down after 2030 for all these metrics.

**Figure 4:**
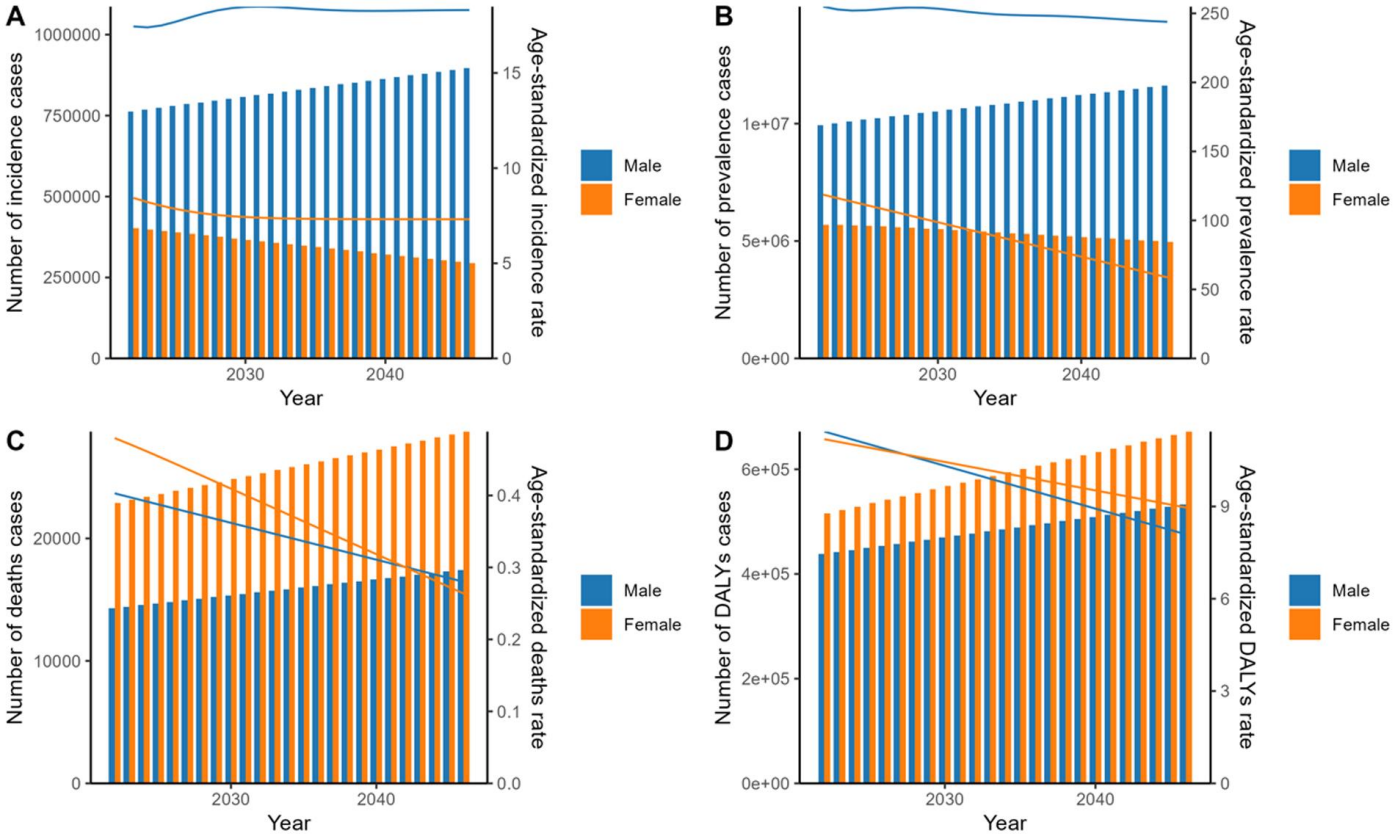
Prediction of age-standardized rate and number of cases of incidence **(A)**, prevalence **(B)**, deaths **(C)**, and DALYs **(D)** by sex using ARIMA model for 2022–2050. Bars represent case counts, while curves depict ASR. DALYs, disability-adjusted life years; ARIMA, autoregressive integrated moving average; ASR, age-standardized rates.

The ARIMA model forecasts trends extending to 2050. As illustrated in the Figure 4 and Supplementary Table S3, While the estimated number of incidence, prevalence, deaths, and DALYs is projected to increase overall from 2022 to 2050, incidence and prevalence are expected to rise in males but decline in females. The ASIR exhibits fluctuations yet maintains a generally stable trajectory, with a projected value of 25.62 by 2050. In contrast, the ASPR is projected to exhibit a general downward trajectory, decreasing from 373.51 in 2022 to 290.89 in 2050. Over the same period, the ASDR has decreased from 0.88 to 0.49, and the ASDALYR has also declined, moving from 22.62 to 16.13.

### Frontier analysis

**Figure 5:**
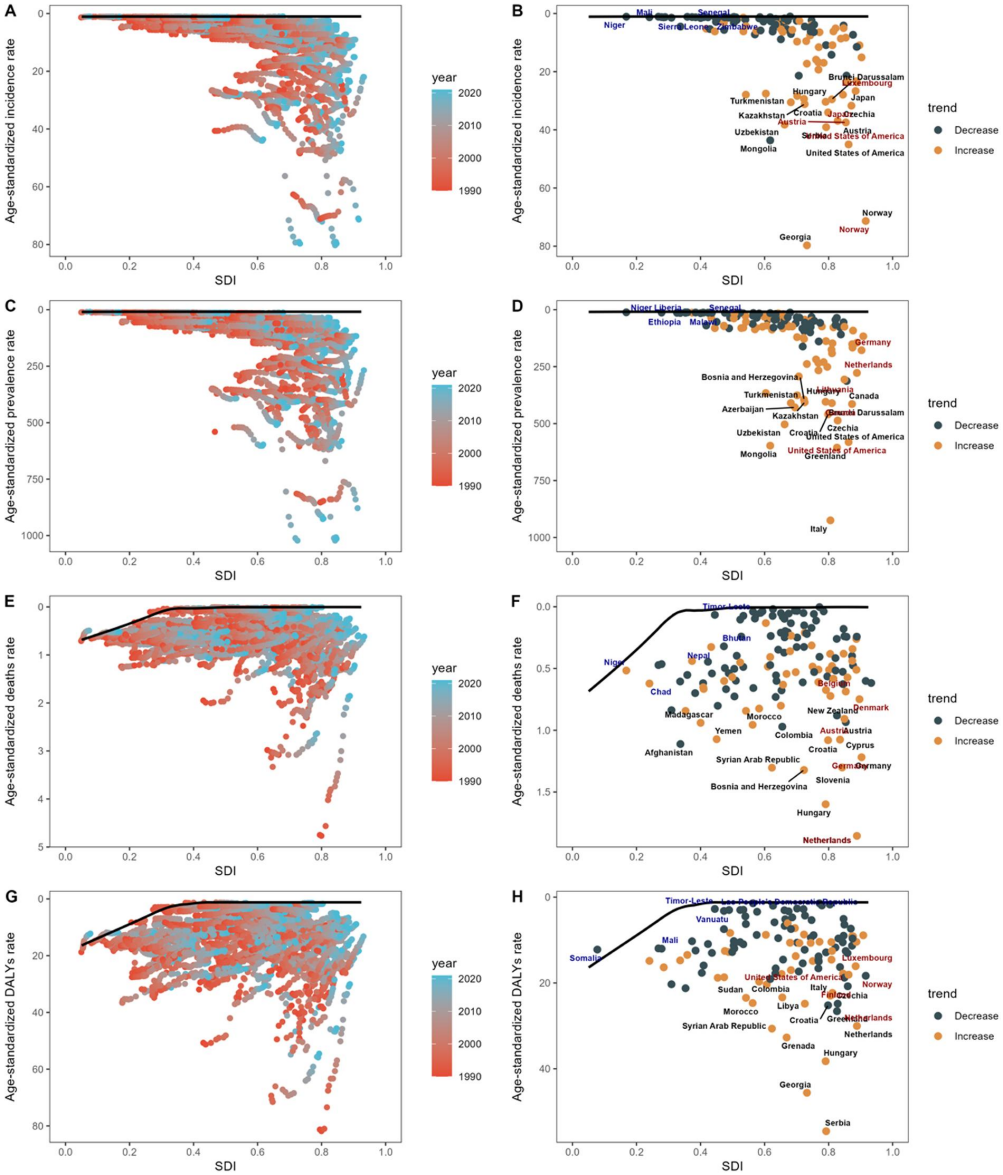
Analysis of the frontier based on the SDI and age-standardized rates of ASIR **(A)**, ASPR **(C)**, ASDR **(E)**, and ASDALYR **(G)** for DMVD from 1990 to 2021 is presented. The frontier is marked in solid black, with countries and territories depicted as dots. A frontier analysis highlighting the relationship between SDI and age-standardized rate of ASIR **(B,D,F,H)** for DMVD in 2021 is also provided. Yellow dots represent an increase from 1990 to 2021, while dark green dots show a decrease in these rates over the same period. SDI, sociodemographic index; DALYs, disability-adjusted life years; ASIR, age-standardized incidence rate; ASPR, age-standardized prevalence rate; ASDR, age-standardized deaths rate; ASDALYR, age-standardized DALYs rate; DMVD, degenerative mitral valve disease.

Focusing on the frontier analysis results from 2021, the visual representations clearly show differences among countries and territories (Figure 5). In terms of ASIR, countries such as Norway, the United States of America, and Georgia have observed large deviations, which have increased compared to previous levels. In contrast, countries like Niger, Mali, and Senegal are closest to the frontier, indicating the best possible outcomes given their SDI conditions. Regarding ASPR, countries including Italy, the United States, and Canada have significantly higher rates, placing them far from the frontier. By comparison, countries like Niger, Liberia, and Ethiopia are closer to the frontier. When assessing ASDR, countries such as the Netherlands, Slovenia, and Hungary have larger gaps from the frontier. Interestingly, high SDI countries like Germany, Denmark, and Australia show relatively high effective differences at their stage of development. Finally, in the analysis of ADSR, Serbia, Georgia, and Hungary are the countries with the largest gaps from the frontier.

### Cross-country inequalities of DMVD

**Figure 6:**
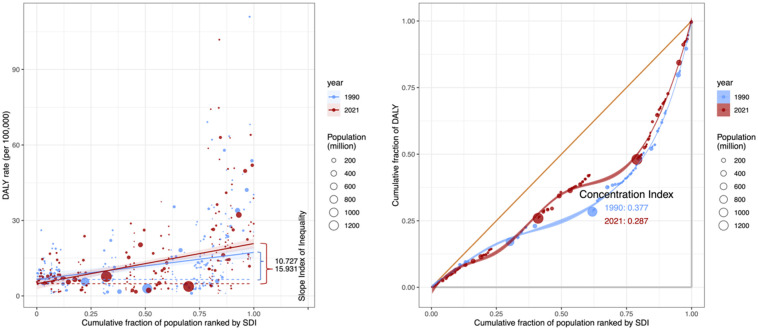
Health inequality regression curves and concentration curves for DMVD in relation to DALYs worldwide, 1990 and 2021. DMVD, degenerative mitral valve disease; val, value; DALYs, disability-adjusted life years; SDI, sociodemographic index.

On a global scale, there are significant absolute and relative inequalities in the ASDALYR of DMVD that are related to the SDI (Figure 6). According to the slope inequality index, countries with higher SDI are shouldering a higher burden. The significant disparity in the ASDALYR for DMVD between nations with the highest and lowest SDI increased from a slope of 10.73 and an intercept of 6.55 in 1990 to a slope of 15.93 and an intercept of 4.87 in 2021. This indicates that the inequality between high SDI and low SDI countries worldwide has been worsening from 1990 to 2021. In comparison, the relative inequality evaluated through the relative concentration index was measured at 0.38 in 1990 and declined to 0.29 in 2019, However, the reduction in relative inequality is evident only in countries with higher SDI, whereas relative inequality is progressing in countries with lower SDI.

## Discussion

DMVD, a principal component of structural heart disease, demands heightened clinical vigilance and targeted policy development due to its evolving epidemiological patterns. Our study outlines the global landscape of DMVD burden across 204 countries and territories, stratified by age, sex, and SDI quintiles from 1990 to 2021. During this period, the overall disease burden of DMVD has risen, posing substantial challenges for healthcare system preparedness in various countries. Prediction for DMVD burden up to 2050 showed that these trend-related changes have not been significantly altered. Frontier analysis reveals significant variations in DMVD burden among countries with similar SDI levels, while the cross-country inequalities are expanding in countries with lower SDI.

DMVD epidemiological indicators change due to various factors. These include education level, economic status, and healthcare accessibility (16). These factors vary significantly across with different development levels. In 2021, the ASIR and ASPR were positively correlated with the SDI. This is primarily because regions with higher SDI levels not only have a higher degree of aging but also possess more advanced healthcare systems and greater patient engagement (17). On the other hand, the ASDR and ASDALYR in low SDI and low-middle SDI regions are astonishingly high, even surpassing that in high-middle SDI regions and close to those in high SDI regions. This is likely attributable to the scarcity of healthcare resources for DMVD and other cardiovascular diseases in lower SDI regions (18). Looking at the development of DMVD disease burden from 1990 to 2021, it is worth noting that there is disparity in the EAPC of ASDR and ASDALYR across different SDI levels, especially between high SDI and low-middle SDI. It demonstrates that in regions with high SDI levels, the continuous investment in healthcare resources for DMVD against the backdrop of an aging population has been remarkably effective. It also suggests that regions with relatively lower SDI may still face a significant shortage of healthcare resources after decades of development (19). The analysis of cross-country inequalities also reflects this point. These findings highlight the significance of prioritizing SDI improvement as a central goal in health-related policies during the development process. In addition to focusing on the development of education and economic status, attention should also be paid to the accessibility of healthcare services for the public. Given the diagnostic and therapeutic challenges of DMVD, it is essential to provide medical assistance for cardiovascular diseases in lower SDI regions.

At the GBD regional level, the analysis found that in 2021, while Central Europe has the highest ASDR and ASDALYR among all regions, the ASDR and ASDALYR in Low Income regions, regions with Minimal Health Systems, and Africa are remarkably high. At the national level, countries with higher levels of development, such as Serbia, Netherlands, and Hungary, although ranking in the top three for ASDR, have shown a downward trend from 1990 to 2021. In contrast, countries such as Bosnia and Herzegovina and Croatia not only have relatively high ASDR but also have shown an upward trend in ASDR from 1990 to 2021. This indicates that there are differences in the formulation and implementation of healthcare policies for DMVD among countries with relatively higher levels of development. It is crucial for these countries to engage in mutual learning and knowledge sharing (20). At the same time, the ASDR in countries with lower levels of development, such as Yemen, Madagascar, and Sudan, was surprisingly high. This indicates that the lack of medical resources in these countries in the field of cardiovascular diseases require the assistance of international organizations (21). These phenomena can also be observed in the ASDALYR results of some countries. Most of these countries were highlighted in the frontier analysis, which indicates that the disease burden of DMVD could have been better managed or alleviated. In general, countries with relatively higher levels of development should focus on the aging process while referring to and learning from the healthcare policies and insurance content of other countries at the same level. When conditions permit, countries with relatively higher levels of development should actively engage in the assistance work for cardiovascular diagnosis and treatment in less developed countries, organized by institutions such as the United Nations and the World Health Organization.

The burden of DMVD shows significant differences in terms of age and gender. In 2021, the ASIR of DMVD demonstrated a gradual increase from age 50, peaking bimodally in the 65–69 and 70–74 age groups. This contrasts with the notable decline among 70–74 age group reported in the 2019 Global Burden of Disease study, potentially reflecting improved living standards and enhanced health awareness in aging populations (5, 22). This suggests that 65 could be an ideal age threshold for cardiac auscultation or ultrasound screening. Early screening facilitates earlier fluid management, pharmacological interventions, and surgical treatments, which may increase the ASIR and ASPR, but could potentially help reduce the ASDR and ASDALYR. Considering the potential impact of the aging process in countries with large population bases, coupled with the relative scarcity of medical resources, the insufficient screening for DMVD is a problem that requires sufficient attention.

Gender analysis revealed a significant disparity. In 2021, females exhibited approximately half the ASIR and ASPR of degenerative mitral valve disease (DMVD) compared to males. This contrasts with certain conventional epidemiological reports, potentially because prior studies often aggregated rheumatic, ischemic, and degenerative types (23–25). This divergence is also likely compounded by two opposing trends. The declining incidence of rheumatic mitral valve disease, which predominantly affects females, and the rising burden of DMVD, which is more common in males. These trends collectively amplified the male-female disparity in 2021. Furthermore, the ASDR in females exceeded that in males, while the ASDALYR in females was almost the same as that in males. Surgical studies suggest that females with mitral regurgitation present with more symptoms at referral, lower rates of valve repair, higher operative mortality, and may have lower postoperative survival rates (26, 27). It is worth pointing out that in the predicted results up to 2050, while the gap in ASIR and ASPR between males and females is expected to widen, the ASDR in females is not expected to be on par with that in males until 2042. Therefore, the worse prognosis in females found by this study should be given full attention and prompt further in-depth clinical research.

This study, based on the latest estimates from GBD 2021, analyzed the global burden of DMVD. However, the study still has some limitations. Firstly, the general limitations of all GBD studies have been described elsewhere. The scarcity of reliable epidemiological data in low- and middle- income countries, as well as diagnostic and other biases in the original studies, also affected the GBD estimates. Secondly, due to the limited definition of DMVD provided by GBD, the disease burden may be underestimated. Finally, our inequality and frontier analyses, while providing valuable insights into disparities, cannot fully incorporate the statistical uncertainty of the source GBD data.

In summary, the number of DMVD's epidemiological indicators has shown an annually increase in overall burden, although the ASR has decreased. Higher SDI countries need to closely monitor changes in domestic DMVD epidemiological indicators and promptly adjust healthcare policies to cope with the aging process. Meanwhile, it is necessary for higher SDI countries to provide medical resource support and cardiology expertise to lower SDI countries. Additionally, this study suggests that 65 is a meaningful age for cardiac auscultation or ultrasound screening. Considering that the ASIR in males is almost twice that in females, while the ASDR is higher in females, an emphasis should be placed on it in screening and treatment. We encourage future studies to build upon this baseline by employing scenario-based modelling, to investigate the potential outcomes under different public health conditions.

## Data Availability

The original contributions presented in the study are included in the article/Supplementary Material, further inquiries can be directed to the corresponding authors.

## Supplementary material

Supplementary Figure S1 The age-standardized rate **(A)** and number of cases **(B)** of incidence, prevalence, deaths, and DALYs due to DMVD by sex, in 2021. Error bars indicate the 95% uncertainty interval (UI). DALYs, disability-adjusted life years; DMVD, degenerative mitral valve disease.

Supplementary Figure S2 The age-standardized rate **(A)** and number of cases **(B)** of incidence, prevalence, deaths, and DALYs due to DMVD by SDI, in 2021. Error bars indicate the 95% uncertainty interval (UI). DALYs, disability-adjusted life years; SDI, sociodemographic index; DMVD, degenerative mitral valve disease.

Supplementary Figure S3 The age-standardized rate **(A)** and number of cases **(B)** of incidence, prevalence, mortality, and DALYs due to DMVD across GBD regions, in 2021. Error bars indicate the 95% uncertainty interval (UI). DALYs, disability-adjusted life years; GBD, global burden of disease; DMVD, degenerative mitral valve disease.

Supplementary Figure S4 Bivariate association between age-standardized rate of DMVD and SDI in 2021, **(A)** ASIR vs. SDI, **(B)** ASPR vs. SDI, **(C)** ASDR vs. SDI, **(D)** ASDALYR vs. SDI. DMVD, degenerative mitral valve disease; SDI, sociodemographic index; DALYs, disability-adjusted life years; ASIR, age-standardized incidence rate; ASPR, age-standardized prevalence rate; ASDR, age-standardized deaths rate; ASDALYR, age-standardized DALYs rate.

Supplementary Figure S5 Bivariate association between age-standardized rates of DMVD and country-level SDI in 2021, (A) ASIR vs. SDI, (B) ASPR vs. SDI, (C) ASDR vs. SDI, (D) ASDALYR vs. SDI. Abbreviations: DMVD, degenerative mitral valve disease; SDI, sociodemographic index; DALYs, disability-adjusted life years; ASIR, age-standardized incidence rate; ASPR, age-standardized prevalence rate; ASDR, age-standardized deaths rate; ASDALYR, age-standardized DALYs rate.

Supplementary Figure S6 Age patterns of the age-standardized rate **(A)** and number of cases **(B)** of incidence, prevalence, deaths, and DALYs due to DMVD at the global level in 2021. Error bars indicate the 95% uncertainty interval (UI). DALYs, disability-adjusted life years; DMVD, degenerative mitral valve disease.

Supplementary Figure S7 Trends in age-standardized rate and number of cases of incidence **(A)**, prevalence **(B)**, deaths **(C)**, and DALYs **(D)** of DMVD from 1990 to 2021. DALYs, disability-adjusted life years; DMVD, degenerative mitral valve disease.

Supplementary Figure S8 Trends in age-standardized rate **(A)** and number of cases **(B)** of incidence, prevalence, mortality, and DALYs of DMVD by sex, from 1990 to 2021. DALYs, disability-adjusted life years; DMVD, degenerative mitral valve disease.

Supplementary Figure S9 Trends in age-standardized rate **(A)** and number of cases **(B)** of incidence, prevalence, deaths, and DALYs of DMVD, by SDI quintiles, from 1990 to 2021. DALYs, disability-adjusted life years; DMVD, degenerative mitral valve disease; SDI, sociodemographic index.

Supplementary Figure 10 Trends in age-standardized rate **(A)** and number of cases **(B)** of incidence, prevalence, deaths, and DALYs of DMVD, by age group, from 1990 to 2021. DALYs, disability-adjusted life years; DMVD, degenerative mitral valve disease.
