# Supplementary figures and images for: The global, regional, and national burden of non-rheumatic degenerative mitral valve disease from 1990 to 2021 and forecast for 2050
Source: Front Cardiovasc Med. 2026 Jan 28;13:1583290. doi: 10.3389/fcvm.2026.1583290 (PMC12892346; doi:10.3389/fcvm.2026.1583290)

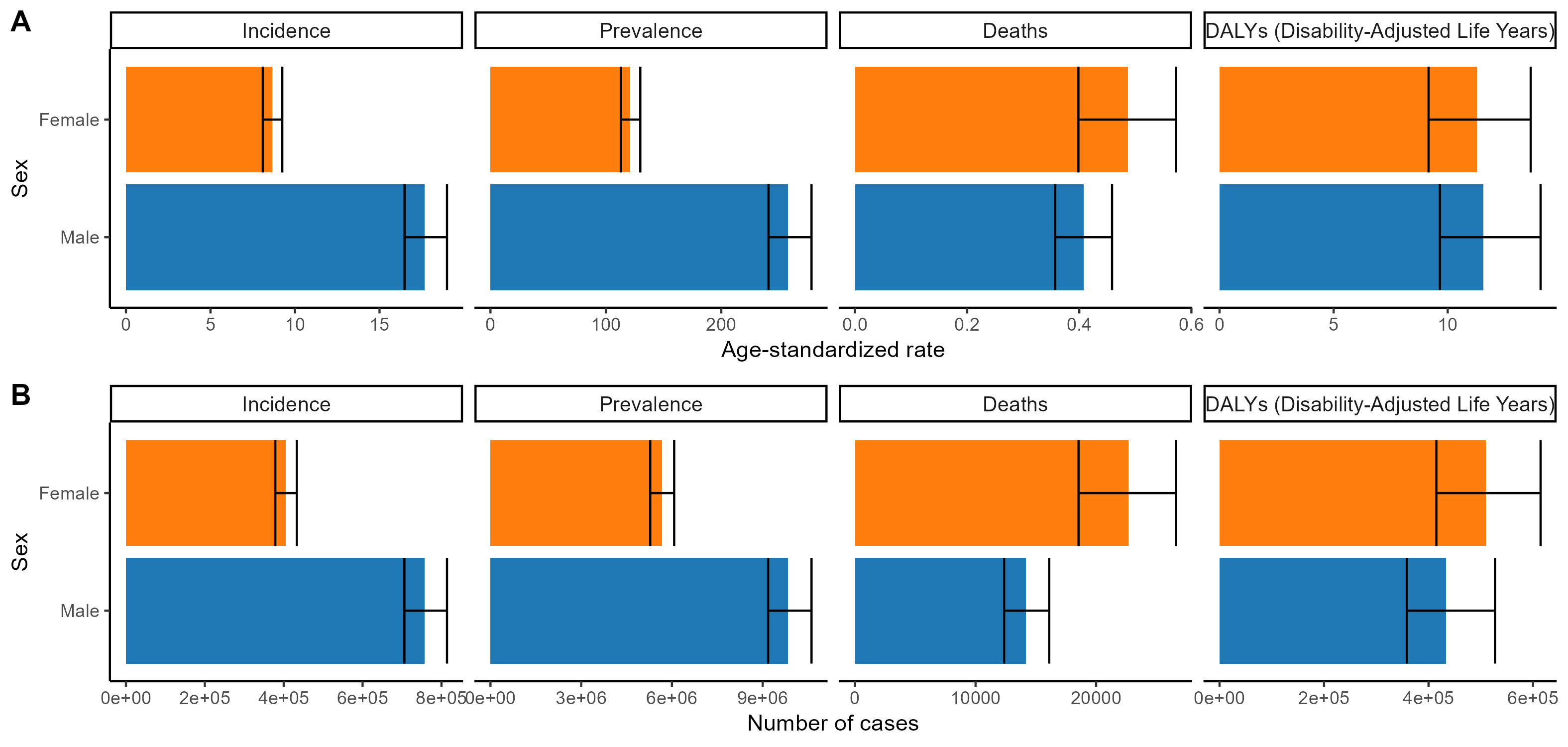

Supplement: Supplementary Figure S1 — The age-standardized rate (A) and number of cases (B) of incidence, prevalence, deaths, and DALYs due to DMVD by sex, in 2021. Error bars indicate the 95% uncertainty interval (UI). DALYs, disability-adjusted life years; DMVD, degenerative mitral valve disease. [file Image1.png]

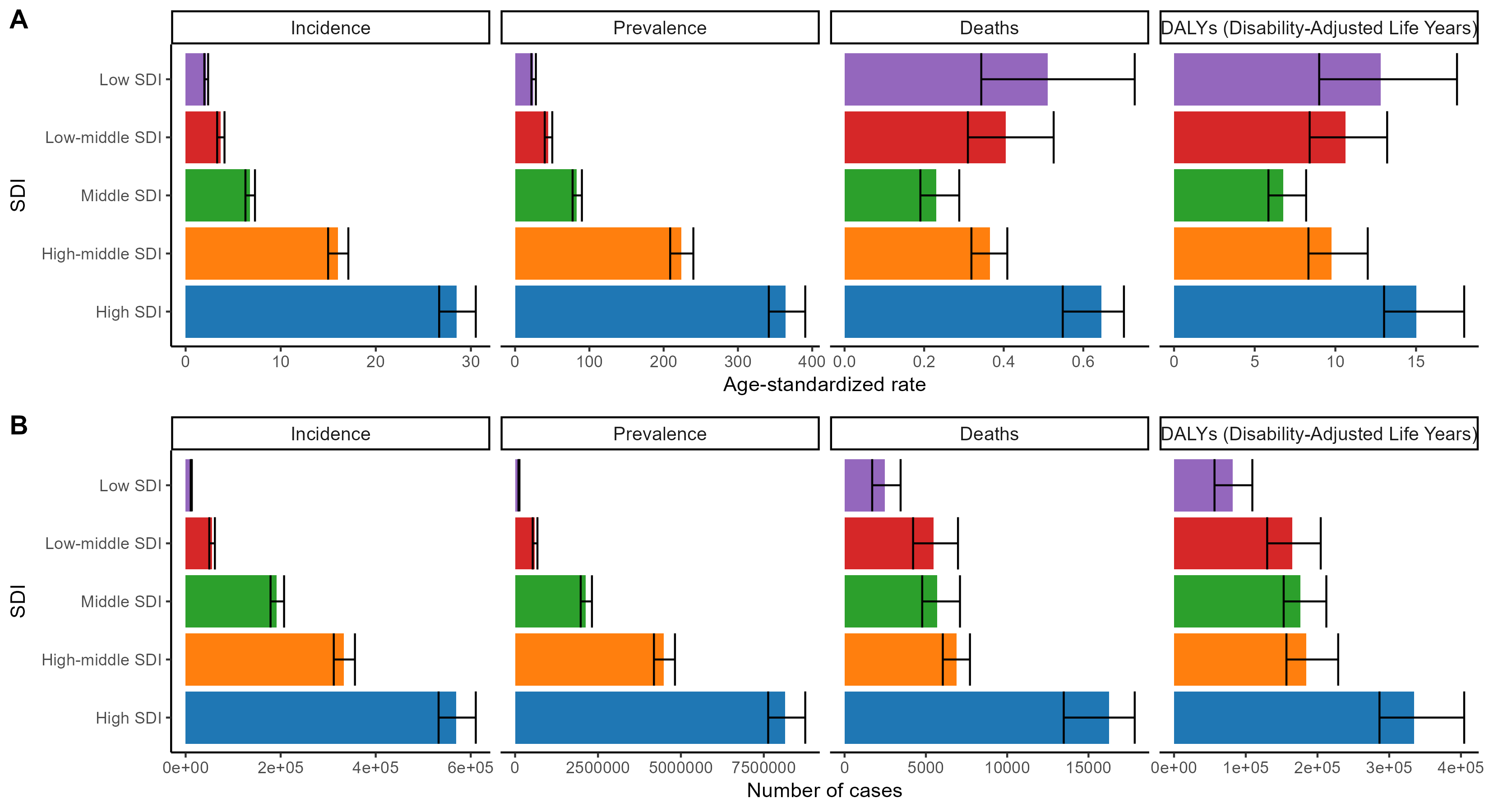

Supplement: Supplementary Figure S2 — The age-standardized rate (A) and number of cases (B) of incidence, prevalence, deaths, and DALYs due to DMVD by SDI, in 2021. Error bars indicate the 95% uncertainty interval (UI). DALYs, disability-adjusted life years; SDI, sociodemographic index; DMVD, degenerative mitral valve disease. [file Image2.png]

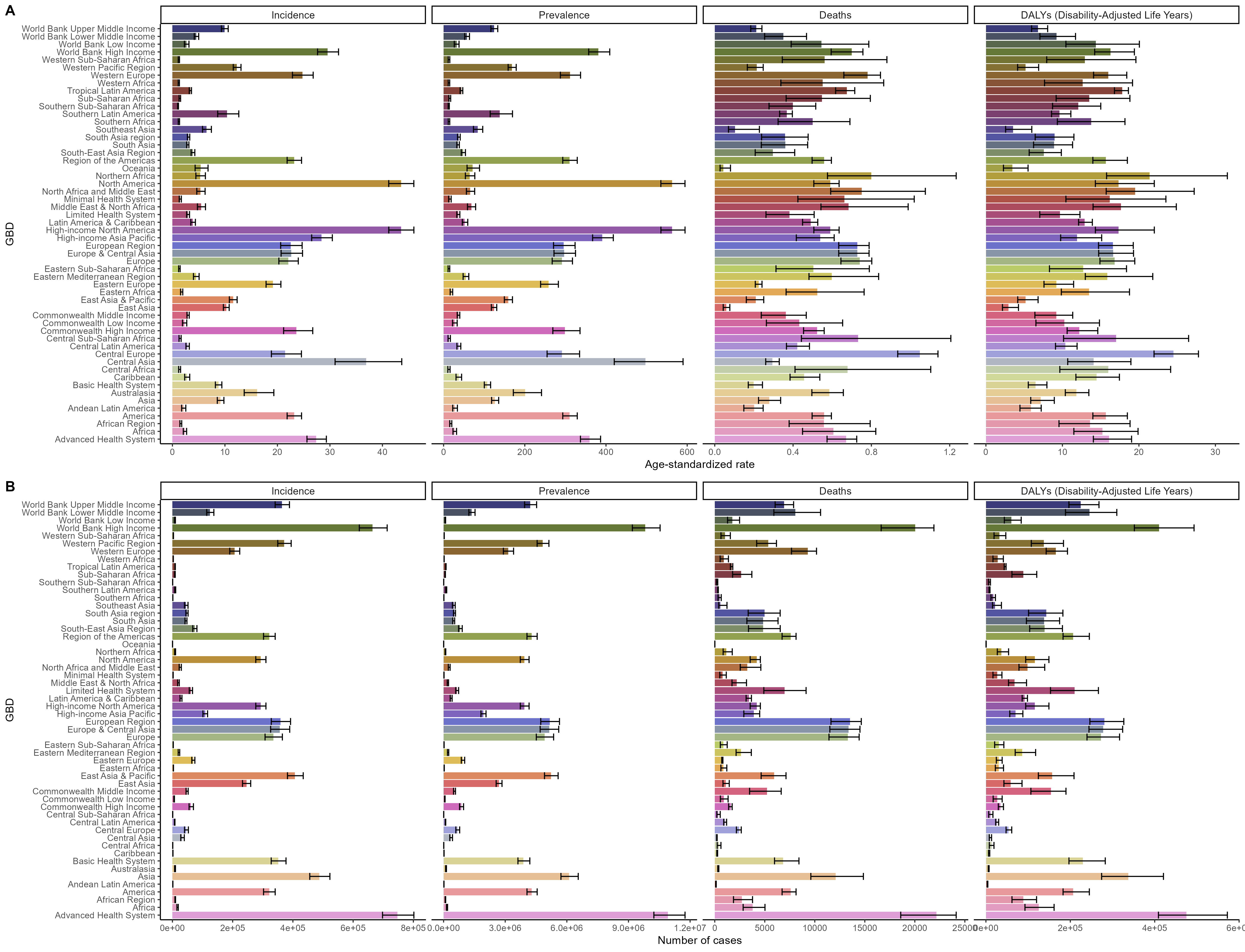

Supplement: Supplementary Figure S3 — The age-standardized rate (A) and number of cases (B) of incidence, prevalence, mortality, and DALYs due to DMVD across GBD regions, in 2021. Error bars indicate the 95% uncertainty interval (UI). DALYs, disability-adjusted life years; GBD, global burden of disease; DMVD, degenerative mitral valve disease. [file Image3.png]

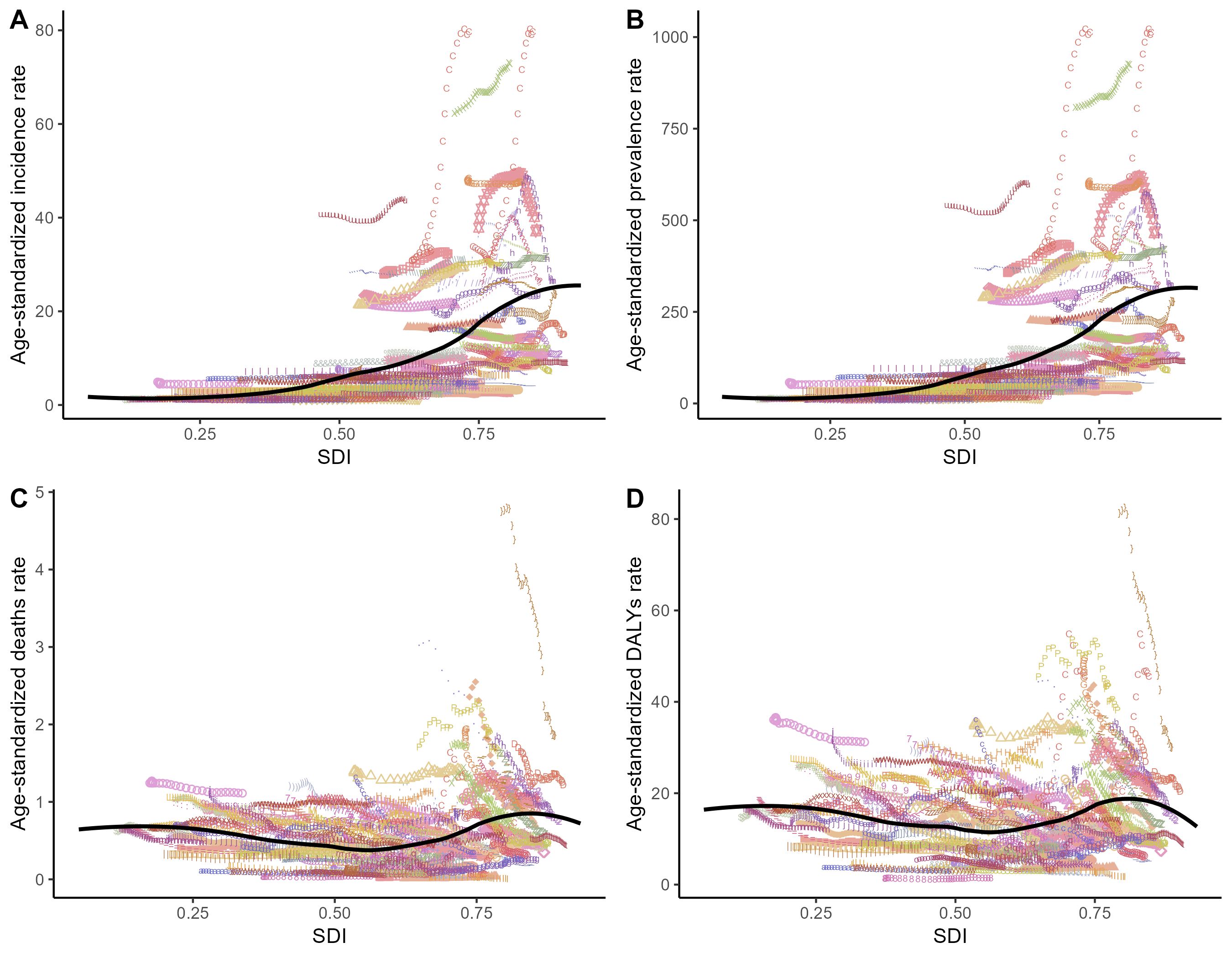

Supplement: Supplementary Figure S4 — Bivariate association between age-standardized rate of DMVD and SDI in 2021, (A) ASIR vs. SDI, (B) ASPR vs. SDI, (C) ASDR vs. SDI, (D) ASDALYR vs. SDI. DMVD, degenerative mitral valve disease; SDI, sociodemographic index; DALYs, disability-adjusted life years; ASIR, age-standardized incidence rate; ASPR, age-standardized prevalence rate; ASDR, age-standardized deaths rate; ASDALYR, age-standardized DALYs rate. [file Image4.png]

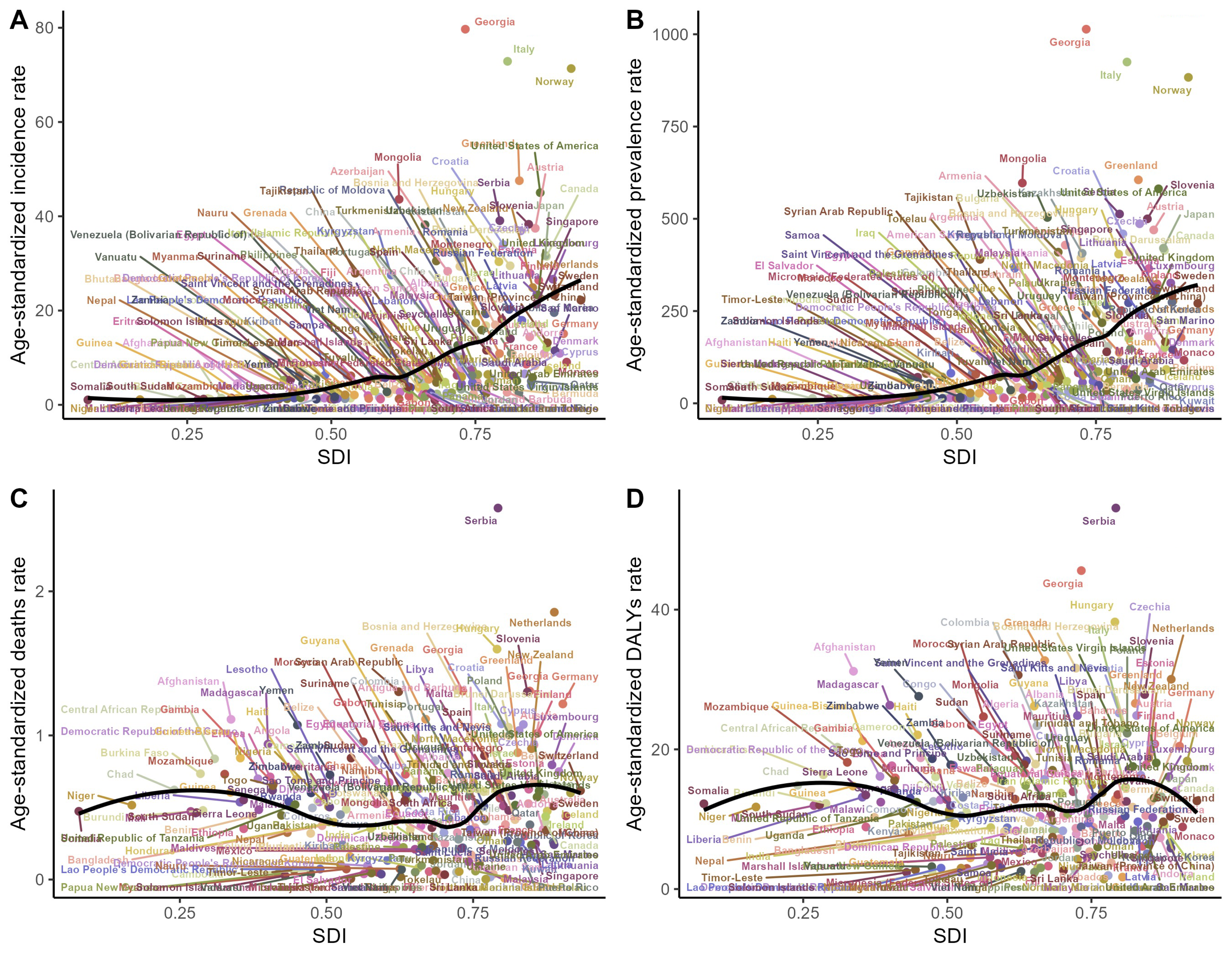

Supplement: Supplementary Figure S5 — Bivariate association between age-standardized rates of DMVD and country-level SDI in 2021, (A) ASIR vs. SDI, (B) ASPR vs. SDI, (C) ASDR vs. SDI, (D) ASDALYR vs. SDI. Abbreviations: DMVD, degenerative mitral valve disease; SDI, sociodemographic index; DALYs, disability-adjusted life years; ASIR, age-standardized incidence rate; ASPR, age-standardized prevalence rate; ASDR, age-standardized deaths rate; ASDALYR, age-standardized DALYs rate. [file Image5.png]

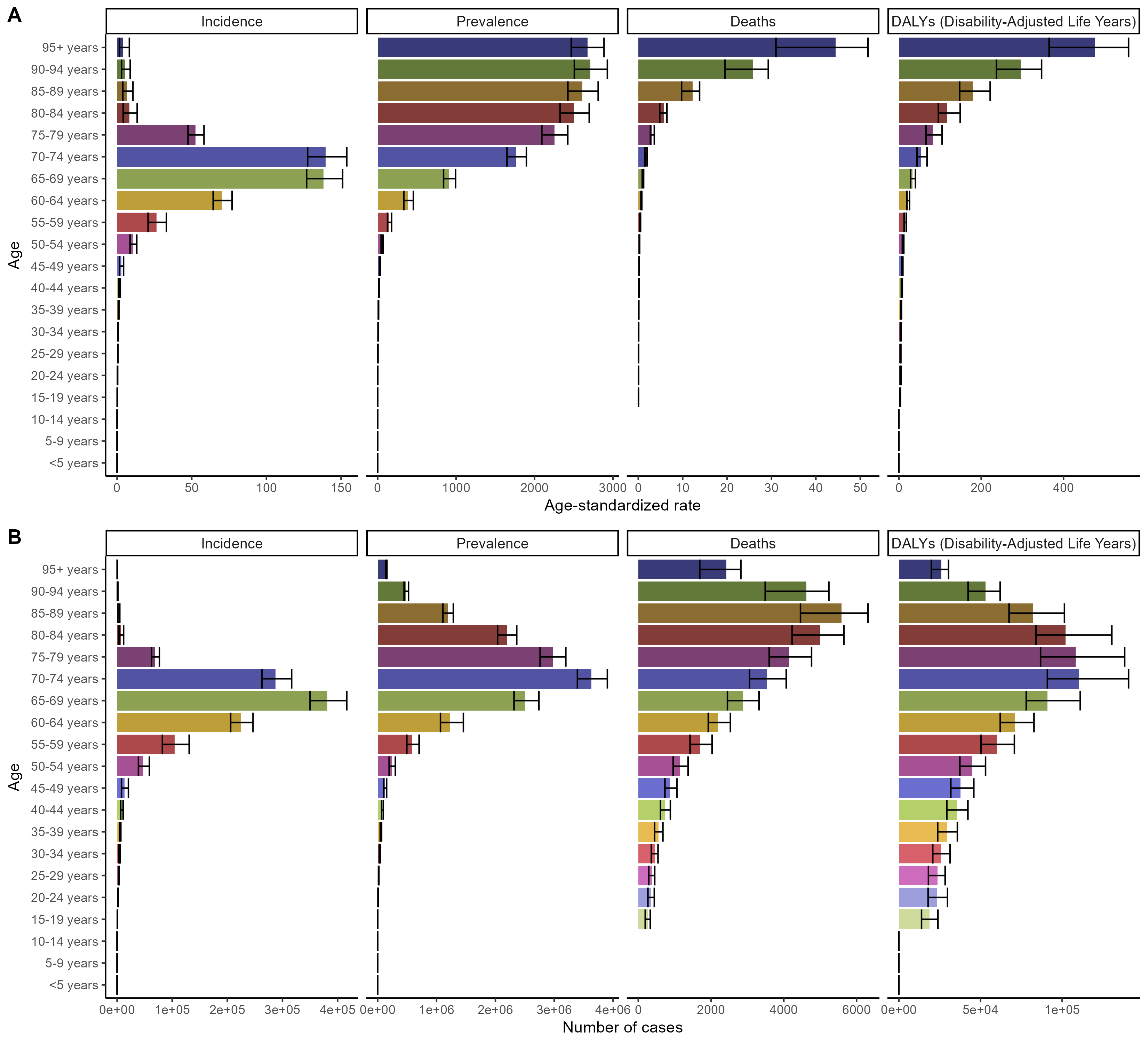

Supplement: Supplementary Figure S6 — Age patterns of the age-standardized rate (A) and number of cases (B) of incidence, prevalence, deaths, and DALYs due to DMVD at the global level in 2021. Error bars indicate the 95% uncertainty interval (UI). DALYs, disability-adjusted life years; DMVD, degenerative mitral valve disease. [file Image6.png]

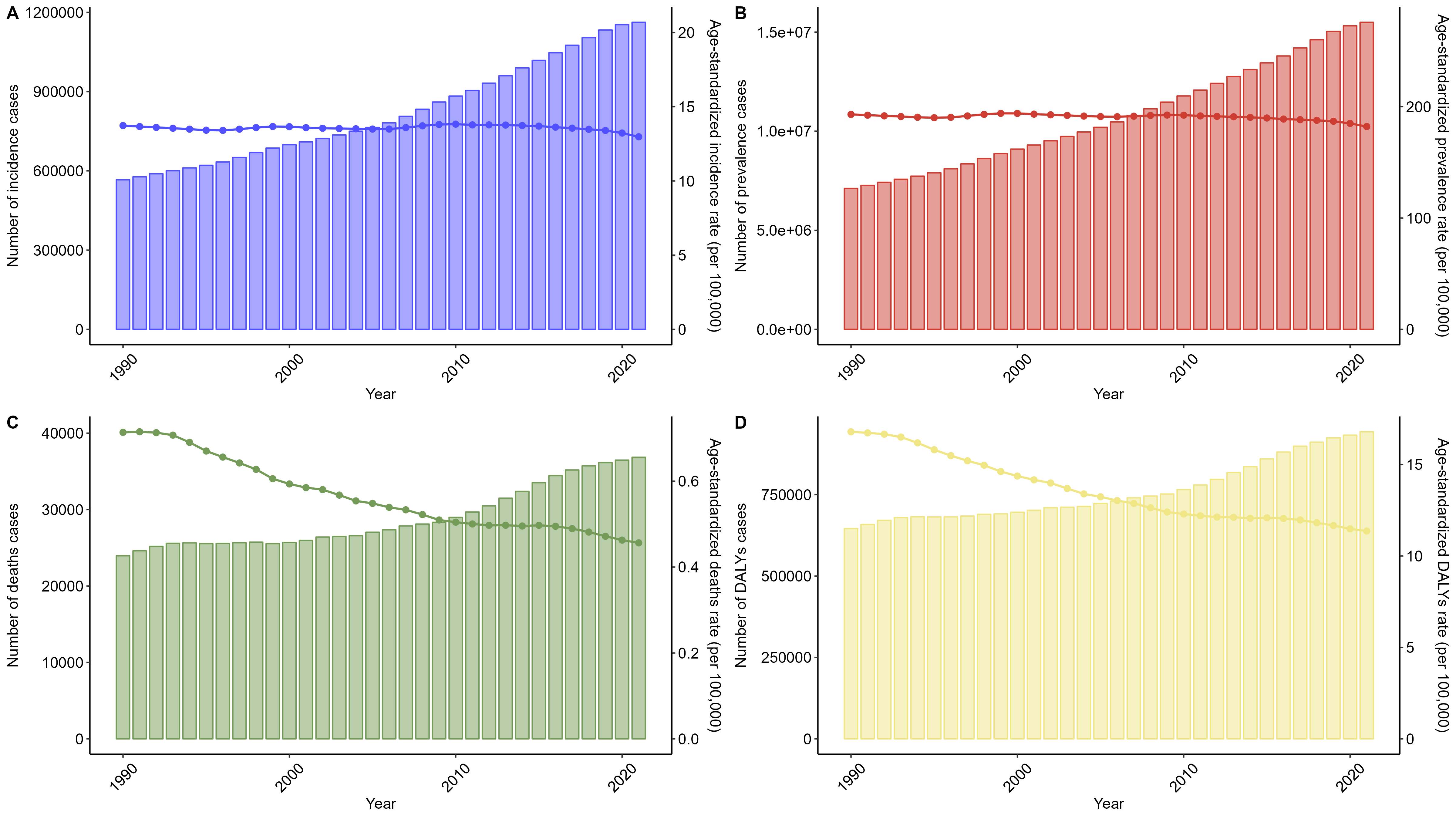

Supplement: Supplementary Figure S7 — Trends in age-standardized rate and number of cases of incidence (A), prevalence (B), deaths (C), and DALYs (D) of DMVD from 1990 to 2021. DALYs, disability-adjusted life years; DMVD, degenerative mitral valve disease. [file Image7.png]

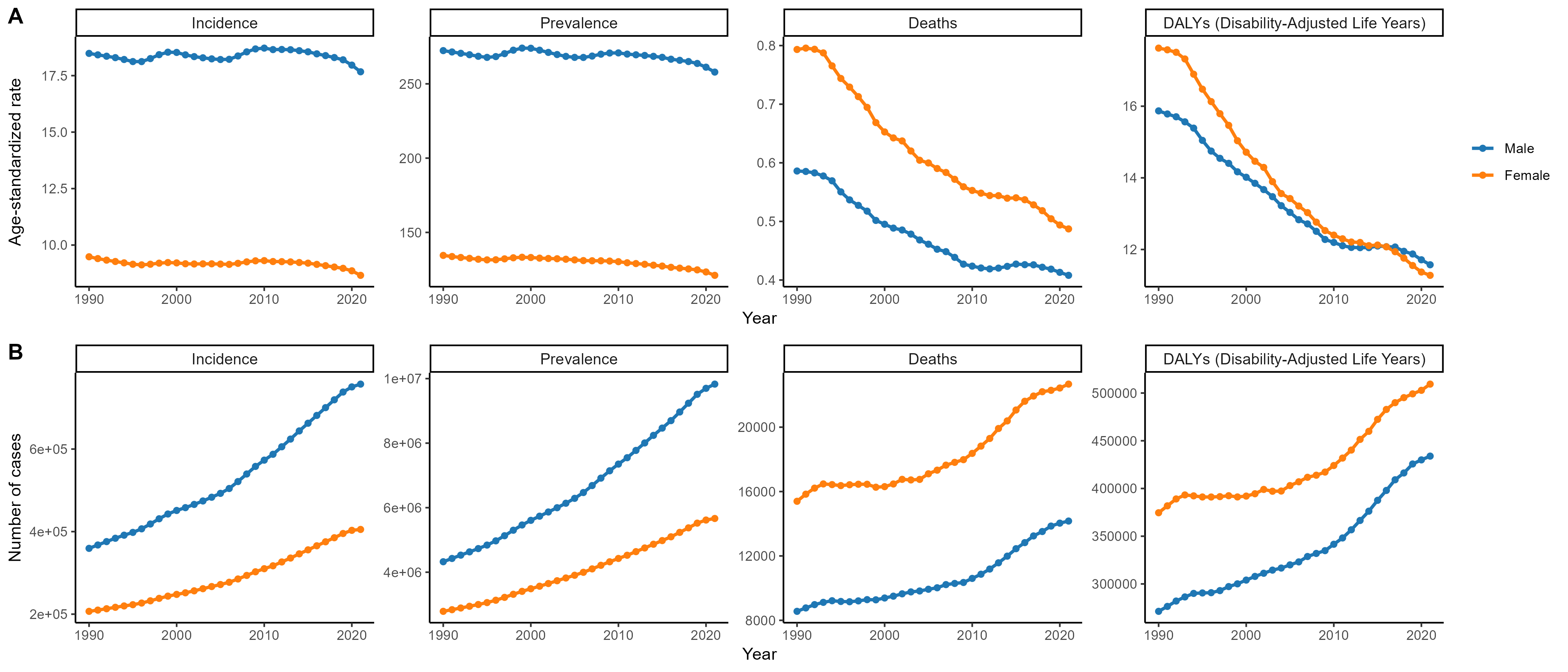

Supplement: Supplementary Figure S8 — Trends in age-standardized rate (A) and number of cases (B) of incidence, prevalence, mortality, and DALYs of DMVD by sex, from 1990 to 2021. DALYs, disability-adjusted life years; DMVD, degenerative mitral valve disease. [file Image8.png]

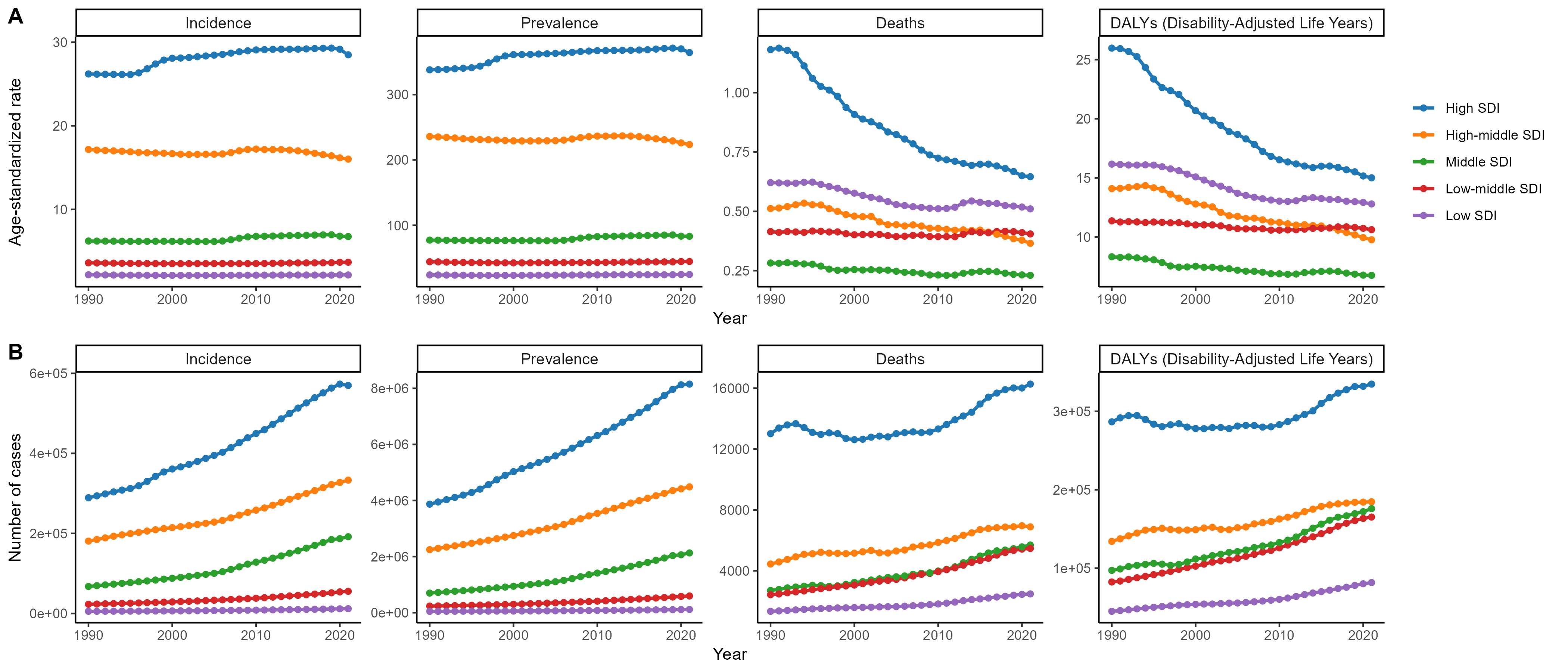

Supplement: Supplementary Figure S9 — Trends in age-standardized rate (A) and number of cases (B) of incidence, prevalence, deaths, and DALYs of DMVD, by SDI quintiles, from 1990 to 2021. DALYs, disability-adjusted life years; DMVD, degenerative mitral valve disease; SDI, sociodemographic index. [file Image9.png]

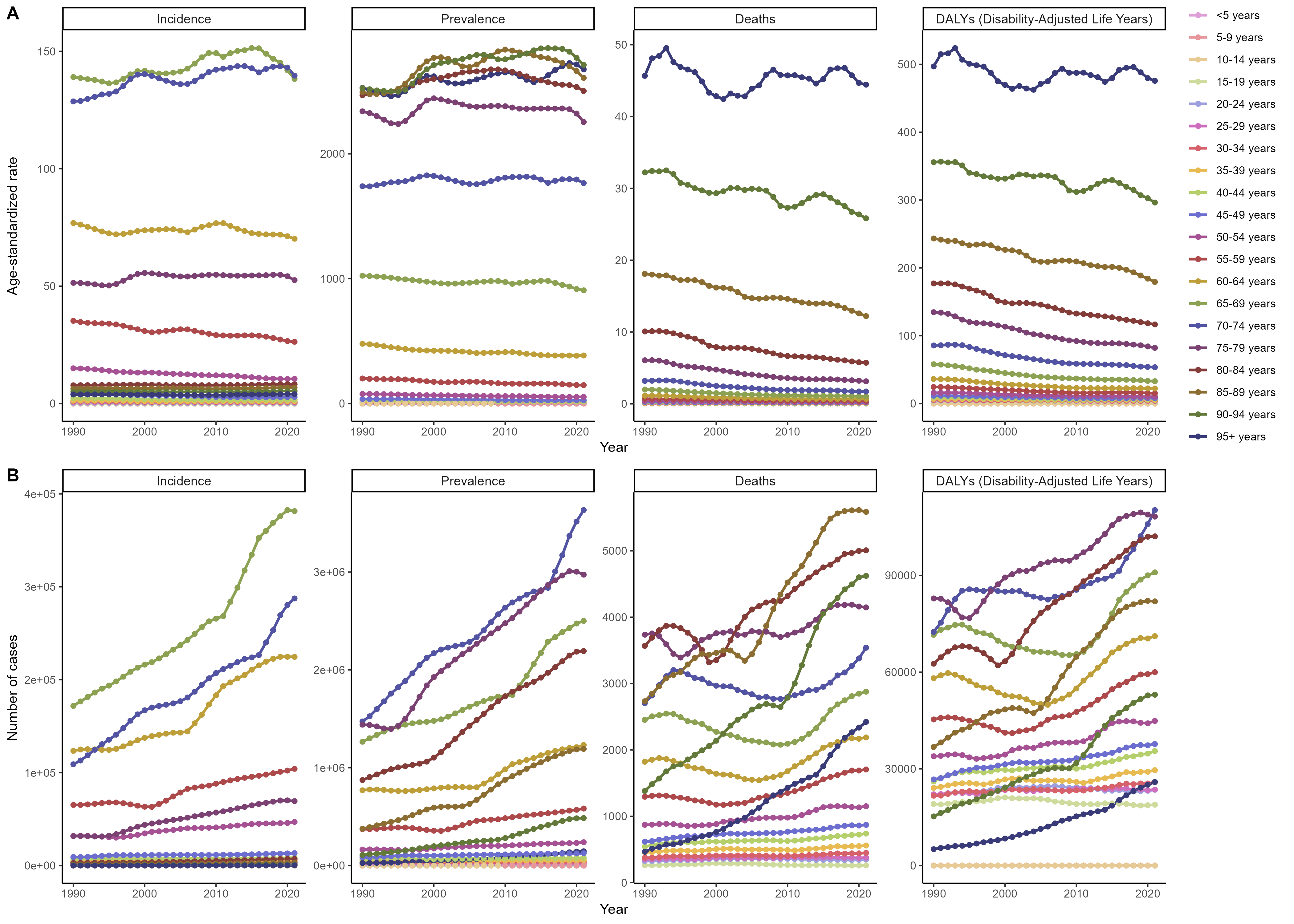

Supplement: Supplementary Figure 10 — Trends in age-standardized rate (A) and number of cases (B) of incidence, prevalence, deaths, and DALYs of DMVD, by age group, from 1990 to 2021. DALYs, disability-adjusted life years; DMVD, degenerative mitral valve disease. [file Image10.png]
